# Early Sensory and Temperament Features in Infants Born to Mothers With Asthma: A Cross-Sectional Study

**DOI:** 10.3389/fpsyg.2021.713804

**Published:** 2021-10-08

**Authors:** Carly A. Mallise, Vanessa E. Murphy, Linda E. Campbell, Alix J. Woolard, Olivia M. Whalen, Gabrielle Milton, Joerg Mattes, Adam Collison, Peter G. Gibson, Frini Karayanidis, Alison E. Lane

**Affiliations:** ^1^School of Psychological Sciences, University of Newcastle, Callaghan, NSW, Australia; ^2^Priority Research Centre GrowUpWell^®^, University of Newcastle, Callaghan, NSW, Australia; ^3^School of Medicine and Public Health, University of Newcastle, Callaghan, NSW, Australia; ^4^Hunter Medical Research Institute, New Lambton Heights, NSW, Australia; ^5^School of Health Sciences, University of Newcastle, Callaghan, NSW, Australia; ^6^Department of Paediatric Respiratory and Sleep Medicine, John Hunter Children’s Hospital, New Lambton Heights, NSW, Australia; ^7^Department of Respiratory and Sleep Medicine, John Hunter Hospital, New Lambton Heights, NSW, Australia; ^8^Priority Research Centre for Healthy Lungs, University of Newcastle, Callaghan, NSW, Australia; ^9^Priority Research Centre for Stroke and Brain Injury, University of Newcastle, Callaghan, NSW, Australia; ^10^Olga Tennison Autism Research Centre, La Trobe University, Bundoora, VIC, Australia

**Keywords:** asthma, mothers, temperament, sensory processing, infancy

## Abstract

Maternal asthma in pregnancy is associated with an increased risk of adverse perinatal outcomes. Adverse perinatal outcomes may result in poorer infant developmental outcomes, such as temperament and sensory difficulties. This study aimed to (1) assess differences in temperament and sensory features between infants born to mothers with and without asthma and (2) investigate differences in these infant behaviours as a function of maternal asthma severity and asthma control. Mothers completed the Carey Temperament Scales and the Sensory Profile 2 at either 6 weeks, 6 months, or 12 months postpartum. Overall, we observed no significant differences between infants born to mothers with and without asthma in their temperament or sensory features; scores in both domains fell within the normative range. More infants in the asthma group, however, were reported to be highly distractible. When compared with normative data, infants in both groups were reported to have poor predictability of biological functions and fewer infants engaged in low levels of sensory behaviours. Some infants were observed to experience difficulties with hyper-reactivity within several domains. Maternal asthma severity and control during pregnancy were not linked to significant differences between infant temperament and sensory features. The present findings indicate that infants born to mothers with asthma are not at an increased risk overall for temperament or sensory difficulties, compared to control infants. However, a subset of infants across both groups may be at risk for attention or sensory hyper-reactivity difficulties. Further research into the developmental outcomes of infants born to mothers with asthma is warranted.

## Introduction

Asthma is the leading respiratory disease to complicate pregnancy, occurring in 8–13% of pregnancies worldwide (Rejnö et al., 2014). Pregnant women with asthma are at an increased risk of adverse perinatal outcomes, including gestational diabetes and pre-eclampsia (Murphy et al., 2011; Wang et al., 2014). Moreover, asthma exacerbations, which occur in as many as 45% of pregnant women with asthma, are associated with an even greater risk of poor infant outcomes, particularly low birth weight and prematurity (Namazy et al., 2013; Robijn et al., 2020). These perinatal outcomes are posited to be due to respiratory alkalosis and hypoxia during pregnancy, resulting in reduced placental blood flow and oxygen supply to the foetus during pregnancy (Meakin et al., 2020). Importantly, such perinatal outcomes confer a higher risk for poor developmental outcomes for infant offspring such as more challenging temperament, greater sensory processing difficulties, increased hyperactivity/inattention, social/peer problems, and developmental delays (Dudova et al., 2014; Guerra et al., 2014; Oudgenoeg-Paz et al., 2017; Cassiano et al., 2020). Children whose mothers have asthma are also at high risk of developing asthma themselves (Lebold et al., 2020), particularly if their mother’s asthma was not managed effectively during pregnancy (Liu et al., 2018). Despite these known risks for adverse outcomes, there is a paucity of research investigating the early behavioural development of infants born to mothers with asthma.

Prenatal maternal immune activation is posited to be one mechanism underlying the relationship between maternal and offspring asthma (Barrett, 2008). Children with asthma have been reported to have more difficulties with cognitive capabilities (e.g., executive functioning; Irani et al., 2017) and behaviour (e.g., attention-deficit/hyperactivity disorder; Kaas et al., 2021). There is also some evidence that children with asthma differ in temperament from the general population (Kim et al., 1980; Lilljeqvist et al., 2002). Further, the relationships between child behaviour and maternal immune activation are potentially mediated by gut microbiome, subsequent to maternal immune status (Cenit et al., 2017; Stokholm et al., 2017). Emerging research indicates that maternal immune activation is also linked to reduced fetal brain growth and poorer developmental outcomes (e.g., Spann et al., 2018). This research suggests maternal immune activation modifies the salience network in the fetal brain, and these changes subsequently influence infant cognition (Boulanger-Bertolus et al., 2018). Infant temperament and sensory features are considered early markers of general developmental and behavioural concerns in childhood and are susceptible to pregnancy complications. For example, recent systematic review and meta-analytic evidence suggests that infants that are born prematurely have poorer attention, higher activity levels, and more auditory processing problems, compared to full-term infants (Bröring et al., 2017; Cassiano et al., 2020). Infants born with a low birthweight have been reported to be fussier and more withdrawn than healthy controls (Honjo et al., 2002; Ohgi et al., 2002). Further, higher proportions of atypical sensory modulation abilities have been reported in toddlers with a low birthweight, compared to the norm (Dudova et al., 2014). However, there is currently no literature examining the temperament and sensory features of infants born to mothers with asthma.

Temperament is defined as the observable, individual differences in behavioural style that emerge early in life (Thomas et al., 1963). Temperament plays an important role in development across the lifespan (e.g., attention and social competence; Abulizi et al., 2017; Tang et al., 2020). Infants with an easy temperament style tend to be positive in mood, high in approach to novelty, quick to adapt, predictable with their biological responses, and low in distractibility (Carey, 1970; Table 1). In contrast, infants with a difficult temperament style tend to be shy, unpredictable in biological functions, harder to sooth, negative in mood, and less adaptable to change. Difficult temperament in infancy and toddlerhood is related to more behavioural problems (e.g., inattention and conduct problems) in later childhood (Abulizi et al., 2017).

**TABLE 1:** Descriptions of the dimensions of temperament.

**Dimension^*a*^**	**Description**
Activity	The infant’s level of motor activity, in relation to the proportion of active/inactive periods during caretaking (e.g., bathing, feeding, and dressing) and play. Higher score indicates more motor activity.
Rhythmicity	The predictability/unpredictability of the infant’s sleep-wake cycle, elimination habits, and feeding pattern. Higher score indicates less predictability.
Approach	The infant’s acceptance or rejection of new situations, objects, or people. Higher score indicates more withdrawal.
Adaptability	The infant’s compliance with change. Higher score indicates slower to adapt.
Intensity	The energy level of an infant’s response, regardless of the quality or direction of the response. Higher score indicates more intense reactions.
Mood	The infant’s amount of positive, happy emotions, in contrast with the amount of negative emotions and crying. Higher score indicates more negative mood.
Distractibility	The effectiveness of stimuli in interfering with the infant’s behaviour. Higher score indicates less distractibility prior to 12 months of age and more distractibility from 12 months onward.
Persistence	The length of time an infant pursues an activity, and the continuation of activities in the face of obstacles. Higher score indicates less persistence.
Threshold	The intensity level of a stimulus needed to evoke a response from the infant. Higher score indicates more sensitivity to sensory stimulation.

*^*a*^Dimensions and descriptions are derived from the framework in Thomas et al. (1963).*

Sensory processing, related to temperament, refers to the child’s behavioural response to their sensory environment. Atypical sensory processing is described as hyper-reactive (e.g., covering ears to unexpected sounds), hypo-reactive (e.g., not responding to a pain stimulus) or sensation seeking (e.g., spinning themselves on a swing beyond the point at which others might become dizzy; Schaaf and Lane, 2015). Children reported to have greater atypical sensory processing features have also been reported as more challenging in their temperament (e.g., negative mood; Li et al., 2020). Further, sensory processing dysfunction can significantly impact daily function and is associated with hyper-activity, inattention, and motor incoordination, which in turn can affect an infant’s ability to engage in play that facilitates development (Dunn, 1997; Sanz-Cervera et al., 2015).

Both temperament and sensory processing can be assessed reliably in infancy, offering an opportunity to explore early bidirectional links between early developmental vulnerabilities and immune function (Engel-Yeger et al., 2014; O’Connor et al., 2017). Infants born to mothers with asthma have been reported to be at-risk for poorer behavioural outcomes (for review, see Whalen et al., 2019), which may be associated with temperament and sensory differences (Ben-Sasson et al., 2013; Mallise et al., 2020). Exploring differences in temperament and sensory processing of infants born to mothers with asthma during the first year of life may allow for the early identification of those at high-risk of poorer developmental and behavioural outcomes in later childhood. In this exploratory study, we aim to (1) characterise the temperament and sensory features of infants born to mothers with asthma in the first year of life, as compared to control infants, and (2) investigate differences in temperament and sensory processing between infants as a function of maternal asthma severity and asthma control during pregnancy. It was hypothesised that infants born to mothers with asthma would have more challenging temperament features and sensory processing difficulties, compared to control infants. It was also hypothesised that infants born to mothers with severe or uncontrolled asthma would have more challenging temperament features and greater sensory processing difficulties, compared to infants born to mothers with mild or moderate, or well- or partly controlled asthma.

## Materials and Methods

A cross-sectional design was used to compare the temperament and sensory features of infants born to mothers with and without asthma at either 6 weeks, 6 months, and 12 months of age. The current study was approved by the Hunter New England Human Research Ethics Committee (reference numbers: 15/05/20/4.05 and 17/12/13/4.01) and the University of Newcastle Human Research Ethics Committee (reference numbers: H-2015-0307 and H-2016-0425).

### Participants

Participants were mothers with (*n* = 187) and without asthma (*n* = 92) and their infant offspring who took part in one of two longitudinal studies on infant development. These studies included visits when infants were 6 weeks, 6 months, and 12 months of age and mothers could enrol into the infant development studies at any time point. Mothers were recruited between May 2015 (May 2017 for control group) and December 2018. For mothers with asthma, involvement in the current study was subsequent to maternal participation in a randomised controlled trial (RCT) of a novel asthma management strategy during pregnancy^1^ (for further details, see Murphy et al., 2016). All mothers provided written, informed consent and were at least 18 years of age. In the asthma group, mothers had a physician diagnosis of asthma, were randomised to asthma intervention between 12- and 22-weeks’ gestation, and had symptoms of asthma or had used asthma treatment in the previous 12 months. Mothers with asthma were excluded if they had a lung disease other than asthma, had used oral corticosteroids for >14 days in the 3 months prior to randomisation, or were attending an antenatal drug and alcohol clinic during pregnancy. Mothers with asthma were further excluded from the infant development study if they were suffering from a severe mental illness at the time of testing. Mothers within the control group were excluded from the current study if they reported a diagnosis of asthma or heavily relied on medical care. Participants were included in the current study regardless of maternal smoking status, maternal medical/psychiatric diagnoses, infant gestational age, or infant birth weight.

### Procedure

Mothers with asthma completed measures of asthma severity and asthma control, preventer and reliever use, and asthma history between 12- and 22-weeks’ gestation, which was obtained from the RCT. Additionally, mothers with and without asthma completed temperament and sensory questionnaires about their infants, at approximately 6 weeks, 6 months, or 12 months of infant age as part of the current study.

#### Maternal Asthma Measures

Adapting the asthma management and prevention guidelines from the Global Initiative for Asthma (2017), mothers were classified into asthma severity and asthma control groups. Severity was assessed based on preventer treatment requirements, while asthma control was based on symptoms and reliever medication use. Thus, asthma severity and asthma control are two separate constructs. It is possible for women with severe asthma to be controlled, and for women with mild asthma to be uncontrolled. More severe asthma indicates greater disease severity, and not necessarily poor disease control. Mothers were classified as having *mild* asthma if they reported using step 1 (reliever alone) or step 2 [low dose inhaled corticosteroids (ICS)] therapy, *moderate* asthma if they were using step 3 [low dose ICS/long-acting beta-antagonist (LABA) or medium/high dose ICS] therapy or *severe* asthma if they were using step 4 (medium/high dose ICS/LABA) therapy. Mothers were classified as having *well-controlled* asthma if they did not experience any of the following symptoms in the previous week: night waking, activity limitation, reliever use >2 times per week, or daytime symptoms >2 times per week. Mothers were classified as having *partly controlled* asthma or *uncontrolled* asthma if 1–2 or 3–4 of these symptoms were present, respectively.

#### Infant Measures

The Carey Temperament Scales (CTS) is a norm-referenced collection of five parent-report questionnaires that measure temperament in children aged from one month to 12 years. The current study used three age-appropriate versions: The Early Infancy Temperament Questionnaire (Medoff-Cooper et al., 1993) at 6 weeks, the Revised Infant Temperament Questionnaire (Carey and McDevitt, 1978) at 6 months and the Toddler Temperament Scales (Fullard et al., 1984) at 12 months. All questionnaires include nine subscales: Activity, Rhythmicity, Approach, Adaptability, Intensity, Mood, Persistence, Distractibility, and Threshold. Items are rated on a 6-point Likert scale, from 1 (*Almost Never*) to 6 (*Almost Always*). Higher scores indicate an infant who is more active, less predictable in their biological functions, more withdrawn, slower to adapt, more intense in reactions, more negative in mood, less persistent, less distractible, and more sensitive to sensory stimulation. Temperament profiles can also be calculated by comparing domain scores to the normative distribution: Scores more than one standard deviation above the normative mean indicate more challenging behaviours while scores more than one standard deviation below the normative mean indicate more manageable behaviours. The CTS has acceptable internal consistency (α ranging from 0.43 to 0.86) and test-retest reliability (*r* ranging from 0.64 to 0.89) and has been validated within clinical populations (Carey and McDevitt, 1978; Fullard et al., 1984; Medoff-Cooper et al., 1993). Within the current study, internal consistency coefficients ranged from 0.34 to 0.84 across the three age groups, for the nine domains.

The Sensory Profile 2 (SP2; Dunn, 2014) is a norm-referenced collection of five parent- and teacher-report questionnaires that assess sensory processing in children (birth to 14 years, 11 months), in relation to everyday sensory events. Two age-appropriate versions were used: the Infant Sensory Profile 2 (ISP2; for 6-week old and 6-month-old infants) and the Toddler Sensory Profile 2 (TSP2; for 12-month-old infants). The SP2 includes eight sensory processing domains: general, auditory, visual, touch, movement, oral, behavioural (TSP2 only), as well as a total score (ISP2 only). Items are rated on a 5-point Likert scale from 5 (*Almost Always*) to 1 (*Almost Never*). High scores indicate that the parent observed the sensory behaviour frequently, whereas low scores indicate the behaviour was observed rarely. The TSP2 can also be scored against four general sensory styles or quadrants: (1) seeking/seeker – how likely an infant is to pursue sensory input; (2) avoiding/avoider – how likely an infant is to withdraw from sensory input; (3) sensitivity/sensor – how likely an infant is to notice sensory input; and (4) registration/bystander – how likely an infant is to miss sensory input. For analyses, we used the total score from the ISP2 and the domain and quadrant scores from the TSP2. Sensory profiles can also be calculated by comparing domain and quadrant scores to the normative distribution. Scores more than one standard deviation above the normative mean indicate more engagement in sensory behaviours while scores more than one standard deviation below the normative mean indicate less engagement in sensory behaviours. The ISP2 and TSP2 have been used with typically developing and clinical samples, with internal consistency coefficients ranging from 0.60 to 0.90 and test-retest reliability coefficients ranging from 0.87 to 0.97 (Dunn, 2014). Within the current study, internal consistency coefficients ranged from 0.34 to 0.75 across the three age groups, for the eight domains.

### Statistical Analysis

The Statistical Package for the Social Sciences (version 25; IBM Corporation, 2017) was used to conduct data analyses in line with the frequentist approach. JASP was used to conduct supplementary Bayesian analyses to examine the strength of evidence under the null hypothesis, as the frequentist approach cannot determine this (JASP Team, 2019; van Doorn et al., 2019). Within our sample, there were four sets of twins within the asthma group; data were randomly selected from one twin of each pair to include in analyses. Participants were included in analyses if they had complete data for the temperament and/or the sensory processing measure, for at least one time-point. Data was analysed cross-sectionally at 6 weeks, 6 months, and 12 months as our small sample sizes did not allow for longitudinal analyses. While this study was cross-sectional, 46% of our total asthma sample and 41.3% of our total control sample provided data for more than one time-point described within this paper. Means and standard deviations were produced for temperament data, sensory processing data, sociodemographic characteristics, and asthma severity and control. Additionally, percentages of each response option were produced for temperament data, sensory processing data, sociodemographic characteristics, as well as maternal asthma severity and control. For the asthma group, independent samples *t*-tests and Chi-squared tests were used to examine differences between participants and non-participants^2^ on asthma severity and asthma control, asthma treatment, body mass index (BMI) and smoking status. Independent samples *t*-tests and Chi-squared tests were used to test for differences between participants in the asthma and control groups on sociodemographic characteristics.

Analysis of Covariance was used to assess for differences in temperament and sensory processing mean scores between infants born to mothers with asthma and control infants, while controlling for gestational age and birth weight. These variables were included as covariates in the analyses because they have been widely reported to be associated with maternal asthma, and infant temperament and sensory outcomes (e.g., Murphy et al., 2011; Dudova et al., 2014; Cassiano et al., 2020). Differences in the distribution of temperament and sensory processing profile scores between infants born to mothers with asthma and control infants were examined using Chi-squared analyses. Differences in temperament and sensory processing mean scores between infants born to mothers with asthma based on asthma severity (mild vs moderate vs severe) and asthma control (well-controlled vs partly controlled vs uncontrolled) were examined using Analysis of Covariance while controlling for gestational age, birth weight, and maternal smoking status. Differences in the distribution of temperament and sensory processing profile scores between infants born to mothers with asthma based on asthma severity (mild vs moderate vs severe) and asthma control (well-controlled vs partly controlled vs uncontrolled) were examined using Chi-squared analyses. As described in van Doorn et al. (2019), Bayes Factors (BF) were used to interpret the strength of evidence using the following guidelines: BF10 1–3 = inconclusive evidence, BF10 3–10 = moderate evidence and BF10 >10 = strong evidence. BF10 that are >1 provide evidence under the alternative hypothesis and BF10 <1 provide evidence under the null hypothesis. For preliminary analyses, level of statistical significance was set to α = 0.05. For main analyses, Bonferroni corrections were applied for multiple comparisons, with alpha levels set to α = 0.006 for all temperament analyses, α = 0.007 for 6 weeks and 6 months sensory processing analyses and α = 0.005 for 12 months sensory processing analyses.

## Results

### Preliminary Statistics

To assess sampling bias within the asthma group, asthma, physical health, and sociodemographic characteristics at entry into the RCT (i.e., during pregnancy; Table 2) were compared based on their participant or non-participant status. There were no statistically significant differences between participant (*n* = 187) and non-participant (*n* = 108) groups on age, parity, asthma severity, asthma control, inhaled corticosteroid use (as asthma preventor medication), BMI or smoking status (all *p* > 0.05). To assess for participation bias, the asthma and control groups were compared on their sociodemographic characteristics (Table 3). Mothers with asthma were (a) younger at infant birth (6 weeks: *p* = 0.001; 6 months: *p* = 0.016); (b) less likely to have an annual household income in the highest bracket (6 months: *p* < 0.001; 6 months: *p* = 0.001); (c) less likely to be born overseas (6 weeks: *p* < 0.001; 6 months: *p* = 0.022); (d) less likely to have a tertiary qualification (6 weeks and 6 months: *p* < 0.001; 12 months: *p* = 0.011); and (e) more likely to have 3 or more children (6 weeks: *p* = 0.019; 6 months: *p* = 0.010; 12 months: *p* = 0.015). Infants born to mothers with asthma were older than infants born to mothers without asthma during the 12-month data collection timepoint (12 months: *p* = 0.002).

**TABLE 2:** Descriptive statistics for asthma and physical health variables between participants and non-participants of the asthma group.

**Asthma/health characteristic**	**Participants (*n* = 187)**	**Non-participants (*n* = 108)**	***p*-Value**
**Age, at infant birth**			
Mean (SD)	30.35 (5.09)	29.89 (5.64)	0.477
Range	20.05–43.94	18.52–41.93	
**Parity, *n* (%)**			
1 child	92 (49.2)	61 (56.5)	0.238
2 children	55 (29.4)	22 (20.4)	
≥3 children	39 (20.9)	21 (19.4)	
Unspecified^*a*^	1 (0.5)	4 (3.7)	
**Body mass index**			
Mean (SD)	30.87 (8.05)	31.18 (8.82)	0.759
Range	19.2–51.4	20.1–61.0	
Underweight	0 (0.0)	0 (0.0)	0.893
Healthy	48 (25.7)	30 (27.8)	
Overweight	56 (29.9)	30 (27.8)	
Obese	81 (43.3)	47 (43.5)	
Unknown^*a*^	2 (1.1)	1 (0.9)	
**Smoking status, *n* (%)**			
Never	107 (57.2)	60 (55.6)	0.952
Ex-smoker	61 (32.6)	37 (34.3)	
Current smoker	18 (9.6)	10 (9.3)	
Unspecified^*a*^	1 (0.5)	1 (0.9)	
**Asthma severity, *n* (%)**			
Mild	111 (59.4)	69 (63.9)	0.756
Moderate	27 (14.4)	15 (13.9)	
Severe	46 (24.6)	23 (21.3)	
Unknown^*a*^	3 (1.6)	1 (0.9)	
**Asthma control, *n* (%)**			
Well-controlled	38 (20.3)	15 (13.9)	0.297
Partly controlled	85 (45.5)	49 (45.4)	
Uncontrolled	62 (33.2)	43 (39.8)	
Unknown^*a*^	2 (1.1)	1 (0.9)	
**ICS use, as asthma preventor treatment^*b*^, *n* (%)**			
Yes	82 (43.9)	45 (41.7)	0.677
No	102 (54.5)	62 (57.4)	
Unspecified^*a*^	3 (1.6)	1 (0.9)	

*t- and Chi-squared-tests were used to determine significant differences between the two groups, and *p*-values are reported here.*

*^*a*^This information was not collected at baseline because mothers either did not attend their appointment or they did not specify the information during their appointment.*

*^*b*^Frequencies/percentages do not add up to total sample size/100% as they are not mutually exclusive.*

**TABLE 3:** Sociodemographic characteristics of the participants in the asthma and control groups at 6 weeks, 6 months, and 12 months of age.

**Sociodemographic characteristic**	**6 weeks**		**6 months**		**12 months**	
	**Asthma**	**Control**	**Asthma**	**Control**	**Asthma**	**Control**
**Sample size, *n***						
Temperament data	142	37	83	50	74	47
Sensory processing data	97	35	57	50	71	47
Total unique dyads	146	37	88	54	74	48
**Maternal age at infant birth (years)**						
Mean (SD)	**30.31 (5.13)**	**33.31 (4.88)**	**30.51 (5.10)**	**32.69 (5.23)**	30.81 (5.15)	32.09 (4.47)
Range	20.05–43.94	22.45–49.83	20.66–43.94	22.45–49.83	21.45–43.94	23.96–49.20
**Infant age, at participation (weeks)**						
Mean (SD)	6.94 (1.77)	6.87 (1.25)	27.89 (2.01)	27.34 (1.61)	**54.51 (3.00)**	**53.01 (1.76)**
Range	3.57–14.86	5.43–11.43	19.86–33.86	24.14–32.00	42.71–64.43	45.57–56.14
**Infant gender, *n* (%)**						
Male	76 (52.1)	17 (45.9)	44 (50.0)	24 (44.4)	39 (52.7)	25 (52.1)
Female	70 (47.9)	20 (54.1)	44 (50.0)	30 (55.6)	35 (47.3)	23 (47.9)
**Maternal country of birth, *n* (%)**						
Australia	**133 (91.1)**	**27 (73.0)**	**78 (88.6)**	**45 (83.3)**	62 (83.8)	43 (89.6)
Overseas	**8 (5.5)**	**10 (27.0)**	**4 (4.5)**	**9 (16.7)**	4 (5.4)	4 (8.3)
Unspecified	5 (3.4)	0 (0.0)	6 (6.8)	0 (0.0)	8 (10.8)	1 (2.1)
**Maternal ethnicity, *n* (%)**						
White	128 (87.7)	15 (40.5)	73 (83.0)	30 (55.6)	64 (86.5)	41 (85.4)
Aboriginal Australian	2 (1.4)	0 (0.0)	3 (3.4)	0 (0.0)	2 (2.7)	0 (0.0)
Asian	3 (2.1)	2 (5.4)	2 (2.3)	3 (5.6)	2 (2.7)	3 (6.3)
Mixed	7 (4.8)	0 (0.0)	4 (4.5)	1 (1.9)	3 (4.1)	1 (2.1)
Other	6 (4.1)	0 (0.0)	5 (5.7)	1 (1.9)	3 (4.1)	0 (0.0)
Unspecified	0 (0.0)	20 (54.1)	1 (1.1)	19 (35.2)	0 (0.0)	3 (6.3)
**Maternal educational attainment, *n* (%)**						
< High school certificate	**23 (15.8)**	**1 (2.7)**	**14 (15.9)**	**1 (1.9)**	**11 (14.9)**	**1 (2.1)**
High school certificate	29 (19.9)	3 (8.1)	**15 (17.0)**	**3 (5.6)**	8 (10.8)	2 (4.2)
Trade qualification	**49 (33.6)**	**4 (10.8)**	21 (23.9)	9 (16.7)	16 (21.6)	8 (16.7)
University degree	**39 (26.7)**	**26 (70.3)**	**31 (35.2)**	**38 (70.4)**	**32 (43.2)**	**35 (72.9)**
Unspecified	26 (70.3)	3 (8.1)	7 (8.0)	3 (5.6)	7 (9.5)	2 (4.2)
**Annual household income^*a*^, *n* (%)**						
0–18,700	**11 (7.5)**	**0 (0.0)**	5 (5.7)	2 (3.7)	5 (6.8)	1 (2.1)
18,701–37,000	13 (8.9)	3 (8.1)	8 (9.1)	3 (5.6)	7 (9.5)	1 (2.1)
37,001–80,000	**42 (28.8)**	**4 (10.8)**	**27 (30.7)**	**7 (13.0)**	17 (23.0)	10 (20.8)
80,001–180,000	64 (43.8)	18 (48.6)	40 (45.5)	28 (51.9)	37 (50.0)	31 (64.6)
180,001 and Over	**8 (5.5)**	**12 (32.4)**	**3 (3.4)**	**13 (24.1)**	3 (4.1)	5 (10.4)
Unspecified	8 (5.5)	0 (0.0)	5 (5.7)	1 (1.9)	5 (6.8)	0 (0.0)
**Parity, *n* (%)**						
1 child	77 (52.7)	17 (45.9)	45 (51.1)	33 (61.1)	**36 (48.6)**	**32 (66.7)**
2 children	**42 (28.8)**	**17 (45.9)**	22 (25.0)	16 (29.6)	21 (28.4)	13 (27.1)
≥3 children	**27 (18.5)**	**1 (2.7)**	**21 (23.9)**	**2 (3.7)**	**17 (23.0)**	**2 (4.2)**
Unspecified	0 (0.0)	2 (5.4)	0 (0.0)	3 (5.6)	0 (0.0)	1 (2.1)
**Maternal physical health condition^*b*^, *n* (%)**						
No	104 (71.2)	30 (81.1)	60 (68.2)	44 (81.5)	48 (64.9)	39 (81.3)
Yes	42 (28.8)	7 (18.9)	28 (31.8)	10 (18.5)	26 (35.1)	9 (18.8)
Cancer	1 (0.7)	0 (0)	1 (1.1)	0 (0)	1 (1.4)	0 (0)
Diabetes	3 (2.1)	0 (0)	3 (3.4)	0 (0)	3 (4.1)	0 (0)
Immune system	7 (4.8)	0 (0)	4 (4.5)	2 (3.7)	4 (5.4)	2 (4.2)
Stomach/gastrointestinal	4 (2.7)	2 (5.4)	2 (2.3)	1 (1.9)	3 (4.1)	0 (0)
Skin	1 (0.7)	0 (0)	2 (2.3)	0 (0)	0 (0)	1 (2.1)
Thyroid	5 (3.4)	1 (2.7)	4 (4.5)	5 (9.3)	4 (5.4)	4 (8.3)
Reproductive system	6 (4.1)	0 (0)	1 (1.1)	2 (3.7)	3 (4.1)	2 (4.2)
Heart	6 (4.1)	2 (5.4)	3 (3.4)	1 (1.9)	4 (5.4)	0 (0)
Neurological	7 (4.8)	0 (0)	6 (6.8)	0 (0)	4 (5.4)	0 (0)
Bone/joint	3 (2.1)	1 (2.7)	2 (2.3)	2 (3.7)	3 (4.1)	1 (2.1)
Ears/nose/throat	2 (1.4)	1 (2.7)	2 (2.3)	1 (1.9)	1 (1.4)	1 (2.1)
Other	7 (4.8)	2 (5.4)	2 (2.3)	2 (3.7)	4 (5.4)	2 (4.2)
**Maternal mental health condition, *n* (%)**						
No	113 (77.4)	29 (78.4)	62 (70.5)	45 (83.3)	51 (68.9)	38 (79.2)
Yes	33 (22.6)	8 (21.6)	26 (29.5)	9 (16.7)	23 (31.1)	10 (20.8)
Depressive disorder	27 (18.5)	6 (16.2)	23 (26.1)	6 (11.1)	17 (23.0)	5 (10.4)
Anxiety disorder	20 (13.7)	4 (10.8)	13 (14.8)	4 (7.4)	12 (16.2)	5 (10.4)
Borderline personality disorder	2 (1.4)	0 (0)	2 (2.3)	0 (0)	1 (1.4)	1 (2.1)

*Cells in bold refer to the statistically significant differences reported in text; α = 0.05.*

*^*a*^Annual household income is reported in AUD.*

*^*b*^Other than asthma.*

### Sample Characteristics

Mothers with asthma had a mean age of 30.3 years (SD = 5.0) when their infants (50.8% male, 97.9% singleton) were born (Table 3). The majority of mothers with asthma were White (86.1%), were Australian born (88.2%) and had at least completed their final year of high school (77%). The median annual household income range for the asthma group was $80,001–180,000. Approximately one quarter of mothers with asthma had a physical and/or a mental health condition (other than asthma). During pregnancy, most mothers with asthma had mild asthma (59.4%), which was partly controlled (45.5%) or uncontrolled (33.2%; Table 2). Within the control group, mothers had a mean age of 32.5 years (SD = 4.6) when their infants (51.1% male, 100% singleton) were born (Table 3). The majority of mothers within the control group were White (87.3%), were Australian born (82.6%) and had obtained a university degree (75%). The median annual household income range for the control group was $80,001–180,000. Approximately 20% of mothers in the control group had a physical and/or a mental health condition.

### Characterisation of Infant Temperament

Mean temperament domain scores of infants born to mothers with asthma and control infants were compared at 6 weeks, 6 months, and 12 months of age (see Supplementary Table 1). The distribution of temperament profiles between infants born to mothers with and without asthma were also compared at each age group (Table 4). When controlling for gestational age and birth weight, there were no statistically significant differences in temperament domain scores between infants born to mothers with asthma and control infants at 6 weeks, 6 months, or 12 months of age (all *p* ≥ 0.006). BF indicated that the strength of evidence was strong for most temperament domains at 6 weeks, 6 months, and 12 months, in favour of the null hypothesis (see Supplementary Table 1). These findings did not change when gestational age and birth weight were not controlled for in analyses (all *p* ≥ 0.006).

**TABLE 4:** Comparison of the distribution of Carey Temperament Scales domain profiles between infants born to mothers with asthma and control infants at 6 weeks, 6 months, and 12 months of age.

**CTS domain**	**Group**	**<2 SD Count (%)**	**< 1SD Count (%)**	**Within 1 SD Count (%)**	**>1 SD Count (%)**	**>2SD Count (%)**	***p*-Value (α = 0.006)**	**BF_10_**	**Direction of evidence**	**Strength of evidence**
Normative distribution		2%	14%	68%	14%	2%				
**6 weeks**										
Activity	Asthma	5 (3.9)	5 (3.9)	93 (72.1)	21 (16.3)	2 (1.6)	0.409	0.010	H_0_	Strong
	Control	0 (0.0)	4 (11.8)	22 (64.7)	8 (23.5)	0 (0.0)				
Rhythmicity	Asthma	4 (2.8)	8 (5.7)	90 (63.8)	26 (18.4)	13 (9.2)	0.795	0.009	H_0_	Strong
	Control	2 (5.4)	2 (5.4)	26 (70.3)	5 (13.5)	2 (5.4)				
Approach	Asthma	7 (4.9)	23 (16.2)	91 (64.1)	19 (13.4)	2 (1.4)	0.125	0.071	H_0_	Strong
	Control	0 (0.0)	7 (20.0)	21 (60.0)	4 (11.4)	3 (8.6)				
Adaptability	Asthma	3 (2.3)	28 (21.9)	85 (66.4)	10 (7.8)	2 (1.6)	0.148	0.055	H_0_	Strong
	Control	0 (0.0)	6 (19.4)	17 (54.8)	7 (22.6)	1 (3.2)				
Intensity	Asthma	9 (6.4)	31 (22.1)	73 (52.1)	23 (16.4)	4 (2.9)	0.578	0.020	H_0_	Strong
	Control	0 (0.0)	10 (27.8)	18 (50.0)	7 (19.4)	1 (2.8)				
Mood	Asthma	9 (6.5)	31 (22.3)	84 (60.4)	12 (8.6)	3 (2.2)	0.047	0.442	H_0_	Inconclusive
	Control	0 (0.0)	3 (8.1)	25 (67.6)	7 (18.9)	2 (5.4)				
Persistence	Asthma	5 (3.6)	30 (21.9)	69 (50.4)	23 (16.8)	10 (7.3)	0.113	0.308	H_0_	Inconclusive
	Control	1 (2.9)	2 (5.7)	21 (60.0)	5 (14.3)	6 (17.1)				
Distractibility	Asthma	22 (16.1)	32 (23.4)	69 (50.4)	10 (7.3)	4 (2.9)	0.106	0.203	H_0_	Moderate
	Control	1 (2.9)	6 (17.6)	22 (64.7)	5 (14.7)	0 (0.0)				
Threshold	Asthma	9 (6.4)	9 (6.4)	88 (62.9)	26 (18.6)	8 (5.7)	0.530	0.020	H_0_	Strong
	Control	0 (0.0)	4 (10.8)	24 (64.9)	7 (18.9)	2 (5.4)				
**6 months**										
Activity	Asthma	2 (2.4)	11 (13.4)	66 (80.5)	2 (2.4)	1 (1.2)	0.105	0.062	H_0_	Strong
	Control	1 (2.0)	11 (22.0)	31 (62.0)	6 12.0)	1 (2.0)				
Rhythmicity	Asthma	0 (0.0)	7 (8.6)	40 (49.4)	23 (28.4)	11 (13.6)	0.197	0.175	H_0_	Moderate
	Control	0 (0.0)	0 (0.0)	28 (57.1)	15 (30.6)	6 (12.2)				
Approach	Asthma	0 (0.0)	4 (5.1)	61 (77.2)	14 (17.7)	0 (0.0)	0.316	0.024	H_0_	Strong
	Control	0 (0.0)	2 (4.1)	35 (71.4)	10 (20.4)	2 (4.1)				
Adaptability	Asthma	0 (0.0)	7 (8.6)	60 (74.1)	10 (12.3)	4 (4.9)	0.758	0.017	H_0_	Strong
	Control	0 (0.0)	6 (12.2)	35 (71.4)	7 (14.3)	1 (2.0)				
Intensity	Asthma	0 (0.0)	5 (6.1)	63 (76.8)	12 (14.6)	2 (2.4)	0.686	0.008	H_0_	Strong
	Control	0 (0.0)	3 (6.0)	41 (82.0)	6 (12.0)	0 (0.0)				
Mood	Asthma	2 (2.4)	17 (20.7)	51 (62.2)	12 (14.6)	0 (0.0)	0.357	0.030	H_0_	Strong
	Control	0 (0.0)	6 (12.0)	37 (74.0)	7 (14.0)	0 (0.0)				
Persistence	Asthma	1 (1.2)	10 (12.0)	55 (66.3)	15 (18.1)	2 (2.4)	0.164	0.026	H_0_	Strong
	Control	0 (0.0)	1 (2.0)	42 (84.0)	6 (12.0)	1 (2.0)				
Distractibility	Asthma	1 (1.2)	17 (20.5)	51 (61.4)	12 (14.5)	2 (2.4)	0.041	0.179	H_0_	Moderate
	Control	0 (0.0)	5 (10.2)	42 (85.7)	1 (2.0)	1 (2.0)				
Threshold	Asthma	0 (0.0)	6 (7.5)	67 (83.8)	6 (7.5)	1 (1.3)	0.356	0.007	H_0_	Strong
	Control	1 (2.1)	2 (4.3)	36 (76.6)	6 (12.8)	2 (4.3)				
**12 months**										
Activity	Asthma	1 (1.4)	10 (14.5)	55 (79.7)	3 (4.3)	0 (0.0)	0.451	0.019	H_0_	Strong
	Control	0 (0.0)	10 (23.8)	29 (69.0)	3 (7.1)	0 (0.0)				
Rhythmicity	Asthma	0 (0.0)	6 (8.1)	55 (74.3)	9 (12.2)	4 (5.4)	0.190	0.061	H_0_	Strong
	Control	0 (0.0)	1 (2.1)	39 (83.0)	7 (14.9)	0 (0.0)				
Approach	Asthma	0 (0.0)	3 (4.2)	61 (84.7)	6 (8.3)	2 (2.8)	0.013	1.705	H_*a*_	Inconclusive
	Control	0 (0.0)	11 (23.4)	30 (63.8)	4 (8.5)	2 (4.3)				
Adaptability	Asthma	0 (0.0)	8 (11.6)	49 (71.0)	12 (17.4)	0 (0.0)	0.568	0.077	H_0_	Strong
	Control	0 (0.0)	3 (6.8)	35 (79.5)	6 (13.6)	0 (0.0)				
Intensity	Asthma	0 (0.0)	13 (18.1)	54 (75.0)	5 (6.9)	0 (0.0)	0.369	0.018	H_0_	Strong
	Control	1 (2.1)	5 (10.6)	39 (83.0)	2 (4.3)	0 (0.0)				
Mood	Asthma	1 (1.4)	5 (6.9)	49 (68.1)	15 (20.8)	2 (2.8)	0.540	0.006	H_0_	Strong
	Control	0 (0.0)	3 (6.4)	38 (80.9)	5 (10.6)	1 (2.1)				
Persistence	Asthma	0 (0.0)	2 (2.9)	55 (78.6)	11 (15.7)	2 (2.9)	0.126	0.142	H_0_	Moderate
	Control	0 (0.0)	1 (2.2)	28 (60.9)	12 (26.1)	5 (10.9)				
Distractibility	Asthma	0 (0.0)	7 (9.6)	60 (82.2)	6 (8.2)	0 (0.0)	0.213	0.152	H_0_	Moderate
	Control	0 (0.0)	2 (4.3)	36 (78.3)	8 (17.4)	0 (0.0)				
Threshold	Asthma	1 (1.4)	8 (11.3)	55 (77.5)	7 (9.9)	0 (0.0)	0.552	0.015	H_0_	Strong
	Control	0 (0.0)	9 (19.1)	33 (70.2)	5 (10.6)	0 (0.0)				

*Chi-square tests were used to determine significant differences between the two groups, and *p*-values are reported here. Bayesian analyses were also conducted to provide the direction and strength of evidence.*

At 6 weeks, 6 months, and 12 months of age, infants from the asthma group and the control group were largely comparable to the normative distribution of temperament scores. However, at 6 weeks of age, infants born to mothers with asthma appeared to be more varied within the domains of intensity, persistence, and distractibility. Of note, approximately 40% of 6-week-old infants born to mothers with asthma were more distractible than the norm, almost double the amount of control infants. This difference in proportions of highly distractible infants between the asthma and control groups was also observed within the 6 months and 12 months groups but with smaller proportions (i.e., 21.7 vs 10.2% at 6 months; 9.6 vs 4.3% at 12 months).

Infants were less varied in their temperament overall at 6 months and 12 months, compared to 6 weeks, with the majority clustering around the normative mean. The exception was the rhythmicity domain at 6 months, where half of the infants born to mothers with asthma, and just over 40% of control infants, fell outside the normal range. At both 6 weeks and 6 months, many infants born to mothers with asthma were reported to have poor predictability of their biological functions (27.6% at 6 weeks; 42% at 6 months), although the proportions were comparable to the control infants at 6 months. This high proportion of arrhythmic infants was not observed in either group at 12 months. Despite the differences in proportions, there were no statistically significant differences in the distribution of temperament profile scores between infants born to mothers with asthma and control infants identified at 6 weeks, 6 months, or 12 months (all *p* ≥ 0.006). BF indicated that the strength of evidence was strong for most temperament domains at 6 weeks, 6 months, and 12 months, in favour of the null hypothesis (Table 4). These results, however, need to be interpreted with caution as the assumption that all expected values are 5 or higher was not met.

### Characterisation of Infant Sensory Features

Mean sensory processing domain scores of infants born to mothers with asthma and control infants were compared at 6 weeks, 6 months, and 12 months of age (see Supplementary Table 2). The distribution of sensory processing profiles between infants born to mothers with and without asthma were also compared at each age group (Table 5). When controlling for gestational age and birth weight, there were no statistically significant differences in sensory processing domain or quadrant scores between infants born to mothers with asthma and control infants at 6 weeks (all *p* > 0.007), 6 months (all *p* > 0.007), or 12 months (all *p* > 0.005). BF indicated that the strength of evidence was strong for most sensory domains at 6 weeks, 6 months, and 12 months, in favour of the null hypothesis (see Supplementary Table 2). These findings did not change when gestational age and birth weight were not controlled for in analyses (all *p* ≥ 0.005).

**TABLE 5:** Comparison of the distribution of Sensory Profile 2 domain and quadrant profiles between infants born to mothers with asthma and control infants at 6 weeks, 6 months, and 12 months of age.

**SP2 domains/quadrant**	**Group**	**<2 SD Count (%)**	**<1 SD Count (%)**	**Within 1 SD Count (%)**	**>1 SD Count (%)**	**>2 SD Count (%)**	***p*-Value**	**BF_10_**	**Direction of evidence**	**Strength of evidence**
Normative distribution		2%	14%	68%	14%	2%				
**6 weeks**										
Total score	Asthma	0 (0.0)	1 (1.0)	80 (82.5)	16 (16.5)	0 (0.0)	0.038	0.174	H_0_	Moderate
	Control	0 (0.0)	2 (5.7)	22 (62.9)	10 (28.6)	1 (2.9)				
**6 months**										
Total score	Asthma	0 (0.0)	0 (0.0)	49 (86.0)	6 (10.5)	2 (3.5)	0.273	0.049	H_0_	Strong
	Control	0 (0.0)	0 (0.0)	47 (94.0)	3 (6.0)	0 (0.0)				
**12 months**										
General	Asthma	1 (1.4)	1 (1.4)	62 (87.3)	5 (7.0)	2 (2.8)	0.832	6.995	H_*a*_	Moderate
	Control	0 (0.0)	0 (0.0)	42 (89.4)	4 (8.5)	1 (2.1)				
Auditory	Asthma	1 (1.4)	0 (0.0)	64 (91.4)	4 (5.7)	1 (1.4)	0.186	0.008	H_0_	Strong
	Control	0 (0.0)	2 (4.2)	39 (81.3)	4 (8.3)	3 (6.3)				
Visual	Asthma	0 (0.0)	3 (4.3)	54 (77.1)	13 (18.6)	0 (0.0)	0.282	0.036	H_0_	Strong
	Control	0 (0.0)	3 (6.5)	31 (67.4)	10 (21.7)	2 (4.3)				
Touch	Asthma	0 (0.0)	0 (0.0)	60 (84.5)	10 (14.1)	1 (1.4)	0.722	0.032	H_0_	Strong
	Control	0 (0.0)	0 (0.0)	37 (78.7)	9 (19.1)	1 (2.1)				
Movement	Asthma	1 (1.4)	0 (0.0)	52 (73.2)	18 (25.4)	0 (0.0)	0.716	0.022	H_0_	Strong
	Control	0 (0.0)	0 (0.0)	35 (74.5)	12 (25.5)	0 (0.0)				
Oral	Asthma	0 (0.0)	0 (0.0)	55 (78.6)	12 (17.1)	3 (4.3)	0.644	0.040	H_0_	Strong
	Control	0 (0.0)	0 (0.0)	40 (85.1)	6 (12.8)	1 (2.1)				
Behavioural	Asthma	0 (0.0)	2 (2.9)	56 (81.2)	6 (8.7)	5 (7.2)	0.640	0.015	H_0_	Strong
	Control	0 (0.0)	2 (4.3)	39 (83.0)	5 (10.6)	1 (2.1)				
Seeking/seeker	Asthma	1 (1.4)	1 (1.4)	53 (74.6)	16 (22.5)	***	0.859	0.004	H_0_	Strong
	Control	0 (0.0)	1 (2.2)	35 (76.1)	10 (21.7)					
Avoiding/avoider	Asthma	0 (0.0)	2 (2.9)	62 (89.9)	4 (5.8)	1 (1.4)	0.861	0.003	H_0_	Strong
	Control	0 (0.0)	1 (2.2)	42 (91.3)	3 (6.5)	0 (0.0)				
Sensitivity/sensor	Asthma	0 (0.0)	0 (0.0)	49 (74.2)	13 (19.7)	4 (6.1)	0.577	0.061	H_0_	Strong
	Control	0 (0.0)	0 (0.0)	38 (82.6)	6 (13.0)	2 (4.3)				
Registration/bystander	Asthma	0 (0.0)	2 (2.9)	62 (89.9)	4 (5.8)	1 (1.4)	0.960	0.005	H_0_	Strong
	Control	0 (0.0)	2 (4.3)	40 (87.0)	3 (6.5)	1 (2.2)				

*Chi-square tests were used to determine significant differences between the two groups, and *p*-values are reported here. Bayesian analyses were also conducted to provide the direction and strength of evidence.*

***No normative scores available for this range.

*Bonferroni correction was used due to multiple comparisons; α = 0.007 at 6 weeks and 6 months, and α = 0.005 at 12 months.*

At 6 weeks, 6 months, and 12 months, scores tended to cluster around the normative mean, for infants in both the asthma and control groups. At 6 weeks and 6 months, the score distributions were skewed to the left. This means that fewer infants were reported to engage in low levels of sensory behaviours. At 12 months, similar distributions were observed, with more infants falling above the normative mean than below. In particular, approximately one quarter of infants from both groups were reported to engage in more movement-based behaviours than the norm. Regarding the sensory quadrants at 12 months, 20–25% of infants from both groups were reported to engage in more sensory-seeking behaviours and have greater sensory sensitivity than the norm. However, there were no statistically significant differences in the distribution of sensory processing profile scores between infants born to mothers with asthma and control infants were identified at 6 weeks, 6 months, or 12 months of age (all *p* > 0.05). BF indicated that the strength of evidence was strong for most sensory domains at 6 weeks, 6 months, and 12 months, in favour of the null hypothesis (Table 5). Again, these results need to be interpreted with caution, as the assumption that all expected values are 5 or higher was not met.

### Comparison of Infant Temperament and Sensory Features Between Maternal Asthma Severity and Control Groups

Mean temperament and sensory processing scores of infants born to mothers with asthma were compared at each age group as a function of maternal asthma severity (mild vs moderate vs severe) and asthma control (well-controlled vs partly controlled vs uncontrolled; see Supplementary Tables 3, 4). After controlling for gestational age, birth weight, and maternal smoking status, there were no statistically significant differences in temperament or sensory processing scores between asthma severity or asthma control groups at 6 weeks, 6 months, or 12 months. These findings did not change when gestational age, birth weight, and maternal smoking status were not controlled for in analyses.

Additionally, no statistically significant differences in the distribution of temperament or sensory processing profile cores between asthma severity or asthma control groups were identified at any age point (see Supplementary Tables 5, 6). BF indicated that the strength of evidence was strong for most temperament and sensory domains at 6 weeks, 6 months, and 12 months, in favour of the null hypothesis (see Supplementary Tables 3–6). Once more, these results need to be interpreted with caution as the assumption that all expected values are 5 or higher was not met.

## Discussion

This is the first study to characterise temperament and sensory processing of infants born to mothers with asthma during pregnancy. We hypothesised that infants born to mothers with asthma would have more challenging temperament features and sensory processing issues, compared to control infants. We also hypothesised that infants born to mothers with severe or uncontrolled asthma would have more challenging temperament features and greater sensory processing issues, compared to infants born to mothers with mild/moderate or well-controlled/partly controlled asthma. Overall, temperament and sensory characteristics of infants born to mothers with asthma did not significantly differ from control infants. Further, there were no significant differences in temperament or sensory features between infants based on asthma severity or asthma control. Thus, our hypotheses were not supported. Results from the Bayesian analyses indicate, for the most part, that there is strong evidence that infants born to mothers with and without asthma do not differ in their temperament or sensory features.

However, there were some findings of potential interest. In relation to temperament, a larger proportion of infants born to mothers with asthma than control infants were highly distractible. This was noted across all three time points, but particularly at 6 weeks. Additionally, infants from both groups were arrhythmic in biological functions at 6 weeks and 6 months. Regarding sensory features, score distributions were narrow and clustered tightly around the normative mean, with mothers of both groups less likely to report that their infants engaged in low levels of sensory behaviours. However, at 12 months, some infants in both groups displayed elevated sensory symptoms in several domains.

### Temperament Profile

At 6 weeks, 6 months, and 12 months of age, infants born to mothers with asthma and control infants were largely comparable to the norm, with the majority clustering around the normative mean. Descriptively, a larger proportion of infants born to mothers with asthma than control infants were highly distractible at all three time points, most notably 6 weeks. Distractibility refers to the infant’s ability to notice and respond to changing environmental stimuli (Medoff-Cooper et al., 1993). In early infancy, high distractibility is interpreted as a positive behaviour because it indicates a high level soothability (Behavioral-Developmental Initiatives, 2007). That is, infants who are more easily distractible are easier to soothe because caregivers can distract them from pain and discomfort. In later infancy, distractibility is more closely associated with attentional abilities (Kannass et al., 2006). Poorer attentional abilities in infancy have been found to persist into later life, emerging as attentional deficits in childhood (Miller et al., 2018), and have been associated with learning difficulties and Attention-Deficit Hyperactivity Disorder (Johnson et al., 2015; Re et al., 2015; West et al., 2018). Nevertheless, child outcomes can be mitigated through early intervention (Feil et al., 2016; Lovett et al., 2017). Screening for the behaviours linked to poorer attention in infancy may help in the early identification of infants at higher risk for poor developmental outcomes.

At 6 weeks and 6 months of age, a large proportion of infants from both cohorts were reported to have a high level of arrhythmia, which refers to the poor predictability of biological functions (e.g., feeding and sleep-wake cycles). Arrhythmia has been reported in approximately 25% of infants in the general population (Cook et al., 2019), which is similar to our sample at the 6 weeks and 12 months timepoints, yet lower than the 42% observed in our sample at 6 months. Arrhythmia in biological functions in infancy has been associated with asthma diagnosis later in childhood (Priel et al., 1990). Infants born to mothers with asthma are at an increased risk of developing asthma themselves (Kashanian et al., 2017). It is not currently known how many infants in our sample will receive an eventual diagnosis of asthma. However, our results show this effect is apparent as early as 4–8 weeks yet may resolve by 12 months of age. To extend upon this preliminary finding, future studies should examine the link between early asthma signs, such as infant wheeze symptoms, and arrhythmia in infancy across the two cohorts. Alternatively, arrhythmia has been linked to differences in infant feeding methods, whereby infants who are breastfed are reported to have more sleep difficulties than bottle-fed infants (Galbally et al., 2013; Kielbratowska et al., 2015). Infant feeding method, rather than asthma, may explain the moderate proportion of arrhythmic infants in our sample, considering that we found similar rates of arrhythmia in our control infants. Despite this, tracking infant biological routines via parent report of temperament may be a useful adjunct to the early care and monitoring of young children with risk for asthma.

### Sensory Profile

At 6 weeks, 6 months, and 12 months of age, infants in both the asthma group and control group showed substantial homogeneity, with scores clustered strongly around the normative mean range. This suggests that our sample had less variability in sensory behaviours than the norm. There also appeared to be a slight negative skew, indicating fewer infants being reported as having low levels of sensory behaviours. Further, at 12 months of age, a large proportion of our sample displayed a higher frequency of behaviours associated with movement hyper-reactivity, seeking and sensitivity. This suggests that proportionally, more of our infants were displaying elevated sensory symptoms across several sensory domains. It is important to note, however, that the published normative data of the ISP2 is based on a US-based sample (*n* = 68), that is smaller than our 6-week Australian-born sample (*n* = 97), so findings must be regarded with caution. Re-norming with a large Australian-based sample may be warranted.

Sensory behaviours characterised by hypersensitivity in early infancy could indicate higher risk for sensory dysfunction later in childhood (Engel-Yeger et al., 2014). Children who tend to be hypersensitive to daily sensory stimuli are observed to have more difficulties in managing changes to daily routines, adapting to new environments, and increased anxiety (Dunn, 1997; Lane et al., 2012). Temperament behaviours such as arrhythmia in biological functions, which were observed to be more prominent in our sample, are closely associated with sensory features including hypersensitivity (Boterberg and Warreyn, 2016).

### Limitations and Directions for Future Research

This study should be considered in light of the following methodological constraints. Firstly, the study was cross-sectional in design, which did not allow for the analysis of changes in temperament and sensory features over time. Thus, any differences in temperament and sensory features between the three age groups may be due to differing samples rather than age. We were not able to conduct longitudinal analyses due to sample size limitations. However, future research into the changes in temperament and sensory features over time in these cohorts would be valuable, considering that differences between the age groups appear to be present. Second, there were several differences in sociodemographic characteristics between participants in the asthma group and the control group. In particular, mothers within the control group were more homogenous in their sociodemographic characteristics, with control mothers overall having a higher level of education and annual income than mothers with asthma. These differences in sociodemographic characteristics between the two groups could explain the (albeit non-significant) difference in proportions of infants who were reported to be highly distractible. It would be worthwhile for future studies with larger samples to conduct more sophisticated analyses with sociodemographic features as covariates, to more closely examine whether infants born to mothers with asthma have a higher level of distractibility. Overall, our findings support future examinations of the early developmental features of infants born to mothers with asthma, as well as the analysis of longer-term developmental outcomes to understand links between early temperament and sensory features and later childhood functioning. Further research may help identify why some infants born to mothers with asthma in our sample presented with temperament and sensory difficulties.

Future studies should also investigate the potential mechanisms underlying the relationship between maternal asthma and infant behavioural outcomes. Gestational age, birth weight, substance use during pregnancy (e.g., tobacco smoking and alcohol consumption), infant feeding method, and socioeconomic status are several factors that may link maternal asthma to our high proportions of distractibility and arrhythmia. However, the exploratory design employed in the current study, coupled with low variance in the sample, did not allow us to undertake more sophisticated analyses to explore the mediating/moderating impact of such factors. We therefore suggest that future research includes these factors in the study design when exploring the link between maternal asthma and infant behaviour. Furthermore, gut microbiome has previously been linked to childhood asthma (Fujimura and Lynch, 2015) and child temperament (Aatsinki et al., 2019). Changes in infant gut microbiome secondary to maternal immune status may result in changes to infant temperament and sensory features, which could be a potential explanation for our finding of high proportions of distractibility and arrhythmia. A recent systematic review (Whalen et al., 2019) highlights the importance of optimal asthma management during pregnancy, and the potential effect this has on child development. Thus, our findings, along with the previous literature, support the need to further investigate the role of maternal asthma in infant behavioural outcomes.

## Conclusion

In this cohort of infants born to mothers with asthma during pregnancy, we observed sensory and temperament features that were mostly comparable to infants born to mothers without asthma at 6 weeks, 6 months, and 12 months of age. Importantly, no differences were observed between asthma and control groups when preterm birth and birth weight were controlled for. However, we found that at all three time points, most prominently at 6 weeks, a larger proportion of infants born to mothers with asthma than control infants were highly distractible. Relative to published norms, infants in both cohorts were arrhythmic in biological functions at 6 weeks and 6 months of age. Additionally, at 12 months of age, some infants in both cohorts displayed elevated sensory symptoms. These findings suggest that a subset of infants in both cohorts may be at risk for developmental difficulties related to temperament (distractibility and arrhythmia) and sensory differences (hypersensitivity) later in childhood. Overall, the findings of the current study indicate that further research into the developmental outcomes of infants born to mothers with asthma is warranted.

## Data Availability Statement

The datasets presented in this article are not readily available because they are being analysed in research outputs yet to be published. Requests to access the datasets should be directed to CM, carly.mallise@uon.edu.au.

## Ethics Statement

The studies involving human participants were reviewed and approved by the Hunter New England Human Research Ethics Committee and the University of Newcastle Human Research Ethics Committee. Written informed consent to participate in this study was provided by the participants’ legal guardian/next of kin.

## Author Contributions

AC, AL, CM, FK, JM, LC, PG, and VM contributed to conception and design of the study. AL, AW, CM, GM, LC, and OW contributed to acquisition of data. AL, CM, and VM contributed to analysis and interpretation of data. AL, AW, CM, GM, and VM contributed to drafting the manuscript. AC, AL, AW, CM, FK, GM, JM, LC, OW, PG, and VM contributed to revising the manuscript. All authors read and approved the final manuscript.

## Funding

CM, AW, and OW were supported by a Research Training Program Stipend Scholarship provided by the Australian Government. VM was supported by a Career Development Fellowship from the National Health and Medical Research Council (1084816). PG was supported by a Practitioner Fellowship from the National Health and Medical Research Council (1155810). The Breathing for Life Trial research project was supported by a National Health and Medical Research Council project grant (1060983). This research was conducted with financial assistance from the Priority Research Centre GrowUpWell^®^ at the University of Newcastle. The publication fees for this manuscript were provided by a Hunter Medical Research Institute Early Career Grant awarded to OW (G2100205). The funding bodies had no role in the design of the study, data collection, data analysis, interpretation of data nor in writing the manuscript.

## Conflict of Interest

The authors declare that the research was conducted in the absence of any commercial or financial relationships that could be construed as a potential conflict of interest.

## Publisher’s Note

All claims expressed in this article are solely those of the authors and do not necessarily represent those of their affiliated organizations, or those of the publisher, the editors and the reviewers. Any product that may be evaluated in this article, or claim that may be made by its manufacturer, is not guaranteed or endorsed by the publisher.

## Footnotes

1 As the randomised controlled trial is still ongoing, treatment allocation group data is not yet available for statistical analyses.

2 Non-participants were mothers with asthma who did not consent to the infant development study or did not have any available temperament or sensory processing data.
