# Supplementary material for: Early Sensory and Temperament Features in Infants Born to Mothers With Asthma: A Cross-Sectional Study
Source: Front Psychol. 2021 Oct 8;12:713804. doi: 10.3389/fpsyg.2021.713804 (PMC8531526; doi:10.3389/fpsyg.2021.713804)
Supplement: Supplementary file 1 [file Table_1.DOCX]

| **Table S1.** Comparison of Carey Temperament Scales data between infants born to mothers with asthma and control infants at 6 weeks, 6 months, and 12 months of age. Analysis of Covariance tests were used to determine significant differences between the two groups, while controlling for gestational age and birth weight; *p*-values are reported here. Bayesian analyses were also conducted to provide the direction and strength of evidence. | | | | | | | |
| --- | --- | --- | --- | --- | --- | --- | --- |
| Age Group | CTS Domain | Asthma  M (SD) | Control  M (SD) | *p*-value (α = 0.006) | BF_10_ | Direction of Evidence | Strength of Evidence |
| 6 weeks |  |  |  |  |  |  |  |
|  | Activity | 3.66 (0.66) | 3.63 (0.62) | .969 | 0.734 | H_0_ | Inconclusive |
|  | Rhythmicity | 3.36 (0.79) | 3.40 (0.78) | .613 | 0.123 | H_0_ | Moderate |
|  | Approach | 2.49 (0.73) | 2.66 (0.81) | .238 | 0.024 | H_0_ | Strong |
|  | Adaptability | 2.31 (0.65) | 2.52 (0.76) | .098 | 0.048 | H_0_ | Strong |
|  | Intensity | 3.79 (0.89) | 3.80 (0.80) | .915 | 0.013 | H_0_ | Strong |
|  | Mood | 2.85 (0.77) | 3.22 (0.72) | .006 | 1.243 | H_a_ | Inconclusive |
|  | Persistence | 2.75 (0.91) | 3.13 (0.93) | .019 | 1.375 | Ha | Inconclusive |
|  | Distractibility | 2.31 (0.75) | 2.51 (0.60) | .122 | 0.036 | H_0_ | Strong |
|  | Threshold | 4.28 (0.65) | 4.33 (0.57) | .661 | 0.022 | H_0_ | Strong |
| 6 months |  |  |  |  |  |  |  |
|  | Activity | 4.27 (0.48) | 4.30 (0.60) | .863 | 0.037 | H_0_ | Strong |
|  | Rhythmicity | 2.80 (0.76) | 2.95 (0.77) | .255 | 0.045 | H_0_ | Strong |
|  | Approach | 2.40 (0.64) | 2.54 (0.70) | .219 | 0.023 | H_0_ | Strong |
|  | Adaptability | 2.16 (0.57) | 2.14 (0.52) | .960 | 0.067 | H_0_ | Strong |
|  | Intensity | 3.59 (0.60) | 3.46 (0.54) | .208 | 0.036 | H_0_ | Strong |
|  | Mood | 2.71 (0.65) | 2.81 (0.56) | .316 | 0.047 | H_0_ | Strong |
|  | Persistence | 3.12 (0.80) | 3.15 (0.62) | .993 | 0.516 | H_0_ | Inconclusive |
|  | Distractibility | 2.19 (0.62) | 2.14 (0.50) | .745 | 0.038 | H_0_ | Strong |
|  | Threshold | 3.85 (0.55) | 3.99 (0.66) | .125 | 1.378 | H_a_ | Inconclusive |
| 12 months |  |  |  |  |  |  |  |
|  | Activity | 3.92 (0.59) | 3.94 (0.64) | .776 | 0.042 | H_0_ | Strong |
|  | Rhythmicity | 2.63 (0.80) | 2.66 (0.58) | .609 | 0.025 | H_0_ | Strong |
|  | Approach | 2.99 (0.82) | 2.89 (0.91) | .844 | 0.051 | H_0_ | Strong |
|  | Adaptability | 3.52 (0.74) | 3.55 (0.65) | .806 | 0.014 | H_0_ | Strong |
|  | Intensity | 3.81 (0.54) | 3.80 (0.62) | .790 | 0.026 | H_0_ | Strong |
|  | Mood | 3.17 (0.63) | 3.09 (0.55) | .718 | 0.043 | H_0_ | Strong |
|  | Persistence | 3.77 (0.64) | 4.10 (0.76) | .013 | 0.458 | H_0_ | Inconclusive |
|  | Distractibility | 4.43 (0.55) | 4.62 (0.55) | .106 | 0.088 | H_0_ | Strong |
|  | Threshold | 3.58 (0.68) | 3.48 (0.75) | .702 | 0.046 | H_0_ | Strong |

Abbreviations: CTS = Carey Temperament Scales; EITQ = Early Infancy Temperament Questionnaire; RITQ = Revised Infant Temperament Questionnaire; TTS = Toddler Temperament Scale.

| **Table S2.** Comparison of Sensory Profile 2 data between infants born to mothers with asthma and control infants at 6 weeks, 6 months, and 12 months of age. Analysis of Covariance tests were used to determine significant differences between the two groups, while controlling for gestational age and birth weight; *p*-values are reported here. Bayesian analyses were also conducted to provide the direction and strength of evidence. | | | | | | | |
| --- | --- | --- | --- | --- | --- | --- | --- |
| Age Group | SP2 Domain/Quadrant | Asthma  M (SD) | Control  M (SD) | *p*-value | BF_10_ | Direction of Evidence | Strength of Evidence |
| 6 weeks |  |  |  |  |  |  |  |
|  | General | 17.62 (3.38) | 19.11 (4.00) | .032 | 0.101 | H_0_ | Moderate |
|  | Auditory | 10.35 (2.39) | 10.73 (2.53) | .444 | 0.015 | H_0_ | Strong |
|  | Visual | 6.85 (2.20) | 7.32 (2.40) | .230 | 0.124 | H_0_ | Moderate |
|  | Touch | 5.41 (2.29) | 5.06 (2.33) | .469 | 0.059 | H_0_ | Strong |
|  | Movement | 4.40 (1.73) | 4.30 (1.71) | .837 | 0.089 | H_0_ | Strong |
|  | Oral | 5.23 (1.47) | 5.03 (1.36) | .433 | 0.022 | H_0_ | Strong |
|  | Total | 54.36 (6.78) | 56.29 (9.14) | .170 | 0.047 | H_0_ | Strong |
| 6 months |  |  |  |  |  |  |  |
|  | General | 15.98 (3.49) | 17.04 (2.47) | .083 | 0.088 | H_0_ | Strong |
|  | Auditory | 11.83 (1.76) | 11.88 (1.60) | .835 | 0.029 | H_0_ | Strong |
|  | Visual | 6.18 (2.33) | 6.23 (2.14) | .774 | 0.023 | H_0_ | Strong |
|  | Touch | 5.66 (2.35) | 4.81 (1.84) | .040 | 0.095 | H_0_ | Strong |
|  | Movement | 8.34 (2.31) | 8.33 (1.48) | .942 | 0.356 | H_0_ | Inconclusive |
|  | Oral | 5.98 (0.57) | 5.75 (0.70) | .030 | 0.568 | H_0_ | Inconclusive |
|  | Total | 53.84 (7.88) | 54.02 (5.23) | .788 | 0.048 | H_0_ | Strong |
| 12 months |  |  |  |  |  |  |  |
|  | General | 16.56 (5.44) | 16.53 (4.20) | .894 | 0.021 | H_0_ | Strong |
|  | Auditory | 10.39 (3.33) | 10.85 (3.73) | .431 | 0.029 | H_0_ | Strong |
|  | Visual | 16.91 (3.45) | 16.89 (4.39) | .736 | 0.057 | H_0_ | Strong |
|  | Touch | 10.38 (2.80) | 10.45 (3.11) | .964 | 0.046 | H_0_ | Strong |
|  | Movement | 18.37 (2.84) | 18.72 (2.01) | .404 | 0.019 | H_0_ | Strong |
|  | Oral | 12.61 (3.78) | 11.74 (3.26) | .232 | 0.033 | H_0_ | Strong |
|  | Behavioural | 11.25 (3.54) | 10.49 (3.18) | .499 | 0.110 | H_0_ | Moderate |
|  | Seeking/Seeker | 30.24 (4.03) | 29.89 (3.35) | .381 | 0.042 | H_0_ | Strong |
|  | Avoiding/Avoider | 15.94 (4.15) | 15.35 (3.26) | .655 | 0.048 | H_0_ | Strong |
|  | Sensitivity/Sensor | 24.61 (6.22) | 23.80 (5.35) | .628 | 0.370 | H_0_ | Inconclusive |
|  | Registration/Bystander | 15.91 (4.01) | 16.13 (4.28) | .709 | 0.019 | H_0_ | Strong |

Note: Bonferroni correction was used due to multiple comparisons; α = .007 at 6 weeks and 6 months, and α = .005 at 12 months.

| **Table S3.** Comparison of temperament and sensory processing mean scores between infants born to mothers with mild, moderate, and severe asthma. Analysis of Covariance tests were used to determine significant differences between the three groups, while controlling for gestational age, birth weight and maternal smoking status; p-values are reported here. Bayesian analyses were also conducted to provide the direction and strength of evidence. | | | | | | | | |
| --- | --- | --- | --- | --- | --- | --- | --- | --- |
| Age | Measure & Domain | Asthma Severity Group | | | *p*-value (α = 0.006) | BF10 | Direction of Evidence | Strength of Evidence |
|  |  | Mild  M (SD) | Moderate  M (SD) | Severe  M (SD) |  |  |  |  |
| 6 weeks |  | *n* = 85 | *n* = 18 | *n* = 38 |  |  |  |  |
|  | CTS - Activity | 3.69 (0.68) | 3.53 (0.62) | 3.61 (0.61) | .483 | 0.040 | H_0_ | Strong |
|  | CTS - Rhythmicity | 3.47 (0.76) | 3.32 (0.92) | 3.12 (0.76) | .101 | 0.064 | H_0_ | Strong |
|  | CTS - Approach | 2.53 (0.74) | 2.38 (0.79) | 2.43 (0.69) | .633 | 0.004 | H_0_ | Strong |
|  | CTS - Adaptability | 2.39 (0.61) | 2.27 (0.77) | 2.14 (0.63) | .137 | 0.015 | H_0_ | Strong |
|  | CTS - Intensity | 3.77 (0.92) | 3.74 (0.95) | 3.74 (0.95) | .780 | 0.008 | H_0_ | Strong |
|  | CTS - Mood | 2.90 (0.82) | 2.86 (0.69) | 2.68 (0.67) | .372 | 0.013 | H_0_ | Strong |
|  | CTS - Persistence | 2.79 (0.91) | 2.72 (0.75) | 2.67 (1.04) | .833 | 0.013 | H_0_ | Strong |
|  | CTS - Distractibility | 2.34 (0.76) | 2.24 (0.68) | 2.23 (0.78) | .741 | 0.005 | H_0_ | Strong |
|  | CTS - Threshold | 4.24 (0.57) | 4.42 (0.54) | 4.28 (0.85) | .238 | 0.008 | H_0_ | Strong |
|  |  | *n* = 58 | *n* = 14 | *n* = 24 |  |  |  |  |
|  | ISP2 – Total Score | 54.53 (7.45) | 55.29 (3.77) | 53.46 (6.67) | .738 | 0.027 | H_0_ | Strong |
| 6 months |  | *n* = 52 | *n* = 11 | *n* = 19 |  |  |  |  |
|  | CTS - Activity | 4.24 (0.48) | 4.55 (0.31) | 4.19 (0.53) | .094 | 0.049 | H_0_ | Strong |
|  | CTS - Rhythmicity | 2.85 (0.76) | 2.63 (0.93) | 2.74 (0.67) | .721 | 0.008 | H_0_ | Strong |
|  | CTS - Approach | 2.38 (0.59) | 2.32 (0.87) | 2.51 (0.64) | .696 | 0.006 | H_0_ | Strong |
|  | CTS - Adaptability | 2.12 (0.50) | 2.14 (0.85) | 2.24 (0.59) | .742 | 0.034 | H_0_ | Strong |
|  | CTS - Intensity | 3.54 (0.59) | 3.70 (0.67) | 3.66 (0.62) | .767 | 0.022 | H_0_ | Strong |
|  | CTS - Mood | 2.72 (0.63) | 2.68 (0.88) | 2.71 (0.59) | .998 | 0.016 | H_0_ | Strong |
|  | CTS - Persistence | 3.10 (0.80) | 3.01 (0.74) | 3.24 (0.86) | .756 | 0.036 | H_0_ | Strong |
|  | CTS - Distractibility | 2.08 (0.55) | 2.23 (0.75) | 2.46 (0.69) | .086 | 0.059 | H_0_ | Strong |
|  | CTS - Threshold | 3.84 (0.54) | 3.78 (0.64) | 3.90 (0.57) | .593 | 0.042 | H_0_ | Strong |
|  |  | *n* = 35 | *n* = 10 | *n* = 11 |  |  |  |  |
|  | ISP2 – Total Score | 53.80 (7.86) | 53.20 (6.97) | 53.91 (9.40) | .776 | 0.019 | H_0_ | Strong |
| 12 months |  | *n* = 45 | *n* = 13 | *n* = 13 |  |  |  |  |
|  | CTS - Activity | 3.98 (0.59) | 3.84 (0.61) | 3.73 (0.66) | .143 | 0.242 | H_0_ | Moderate |
|  | CTS - Rhythmicity | 2.71 (0.83) | 2.59 (0.91) | 2.45 (0.64) | .501 | 0.018 | H_0_ | Strong |
|  | CTS - Approach | 3.00 (0.81) | 2.92 (1.00) | 2.89 (0.60) | .760 | 0.021 | H_0_ | Strong |
|  | CTS - Adaptability | 3.63 (0.69) | 3.20 (0.96) | 3.43 (0.58) | .172 | 0.025 | H_0_ | Strong |
|  | CTS - Intensity | 3.82 (0.56) | 3.76 (0.51) | 3.81 (0.62) | .943 | 0.025 | H_0_ | Strong |
|  | CTS - Mood | 3.22 (0.65) | 3.12 (0.74) | 3.03 (0.45) | .528 | 0.036 | H_0_ | Strong |
|  | CTS - Persistence | 3.82 (0.62) | 3.87 (0.66) | 3.60 (0.73) | .595 | 0.015 | H_0_ | Strong |
|  | CTS - Distractibility | 4.47 (0.55) | 4.28 (0.58) | 4.40 (0.47) | .648 | 0.043 | H_0_ | Strong |
|  | CTS - Threshold | 3.53 (0.76) | 3.74 (0.53) | 3.56 (0.57) | .863 | 0.038 | H_0_ | Strong |
|  |  | *n* = 42 | *n* = 13 | *n* = 13 |  |  |  |  |
|  | TSP2 - General Processing | 16.60 (5.08) | 17.15 (7.93) | 16.23 (3.98) | .997 | 0.008 | H_0_ | Strong |
|  | TSP2 - Auditory Processing | 10.00 (2.92) | 11.38 (5.32) | 10.62 (2.33) | .588 | 0.030 | H_0_ | Strong |
|  | TSP2 - Visual Processing | 17.31 (3.48) | 16.17 (4.24) | 17.00 (2.45) | .534 | 0.016 | H_0_ | Strong |
|  | TSP2 - Touch Processing | 10.17 (2.88) | 11.15 (2.97) | 9.85 (2.51) | .709 | 0.073 | H_0_ | Strong |
|  | TSP2 - Movement Processing | 18.79 (2.43) | 17.08 (4.31) | 17.69 (1.80) | .058 | 0.172 | H_0_ | Moderate |
|  | TSP2 - Oral Processing | 12.80 (3.93) | 12.69 (4.09) | 11.85 (3.24) | .779 | 0.058 | H_0_ | Strong |
|  | TSP2 - Behavioural Processing | 11.65 (3.45) | 10.77 (4.44) | 10.08 (2.50) | .361 | 0.051 | H_0_ | Strong |
|  | TSP2 - Seeking/Seeker Quadrant | 30.90 (4.03) | 28.08 (5.14) | 30.69 (1.93) | .036 | 0.321 | H_0_ | Inconclusive |
|  | TSP2 - Avoiding/Avoider Quadrant | 15.95 (4.22) | 16.92 (5.31) | 15.46 (2.96) | .990 | 0.027 | H_0_ | Strong |
|  | TSP2 - Sensitivity/Sensor Quadrant | 25.13 (6.19) | 24.77 (7.62) | 22.62 (4.77) | .726 | 0.079 | H_0_ | Strong |
|  | TSP2 – Registration/Bystander Quadrant | 15.43 (3.82) | 17.18 (5.65) | 16.23 (3.11) | .525 | 0.020 | H_0_ | Strong |

CTS = Carey Temperament Scales; ISP2 = Infant Sensory Profile 2; TSP2 = Toddler Sensory Profile 2.

| **Table S4.** Comparison of temperament and sensory processing mean scores between infants born to mothers with well-controlled, partly-controlled, and uncontrolled asthma. Analysis of Covariance tests were used to determine significant differences between the three groups, while controlling for gestational age, birth weight and maternal smoking status; p-values are reported here. Bayesian analyses were also conducted to provide the direction and strength of evidence. | | | | | | | | |
| --- | --- | --- | --- | --- | --- | --- | --- | --- |
| Age | Measure & Domain | Asthma Control Group | | | *p*-value (α = 0.006) | BF_10_ | Direction of Evidence | Strength of Evidence |
|  |  | Well  M (SD) | Partly  M (SD) | Un  M (SD) |  |  |  |  |
| 6 weeks |  | *n* = 30 | *n* = 68 | *n* = 44 |  |  |  |  |
|  | CTS - Activity | 3.69 (0.59) | 3.60 (0.70) | 3.73 (0.65) | .829 | 0.055 | H_0_ | Strong |
|  | CTS - Rhythmicity | 3.29 (0.79) | 3.36 (0.75) | 3.41 (0.85) | .670 | 0.009 | H_0_ | Strong |
|  | CTS - Approach | 2.45 (0.81) | 2.37 (0.64) | 2.65 (0.74) | .074 | 0.039 | H_0_ | Strong |
|  | CTS - Adaptability | 2.38 (0.60) | 2.15 (0.58) | 2.51 (0.74) | .029 | 0.042 | H_0_ | Strong |
|  | CTS - Intensity | 3.88 (0.89) | 3.72 (0.88) | 3.80 (0.91) | .741 | 0.006 | H_0_ | Strong |
|  | CTS - Mood | 2.95 (0.72) | 2.66 (0.65) | 3.04 (0.91) | .053 | 0.044 | H_0_ | Strong |
|  | CTS - Persistence | 2.87 (1.06) | 2.65 (0.86) | 2.83 (0.88) | .674 | 0.014 | H_0_ | Strong |
|  | CTS - Distractibility | 2.21 (0.78) | 2.19 (0.67) | 2.53 (0.82) | .051 | 0.037 | H_0_ | Strong |
|  | CTS - Threshold | 4.29 (0.60) | 4.20 (0.68) | 4.38 (0.63) | .410 | 0.005 | H_0_ | Strong |
|  |  | *n* = 16 | *n* = 47 | *n* = 33 |  |  |  |  |
|  | ISP2 – Total Score | 51.56 (5.90) | 54.30 (6.78) | 55.42 (6.73) | .173 | 0.019 | H_0_ | Strong |
| 6 months |  | *n* = 15 | *n* = 43 | *n* = 25 |  |  |  |  |
|  | CTS - Activity | 4.32 (0.42) | 4.25 (0.39) | 4.27 (0.63) | .721 | 0.010 | H_0_ | Strong |
|  | CTS - Rhythmicity | 2.54 (0.70) | 2.92 (0.78) | 2.75 (0.74) | .181 | 0.019 | H_0_ | Strong |
|  | CTS - Approach | 2.32 (0.48) | 2.49 (0.65) | 2.31 (0.70) | .423 | 0.009 | H_0_ | Strong |
|  | CTS - Adaptability | 1.99 (0.37) | 2.23 (0.65) | 2.13 (0.53) | .285 | 0.091 | H_0_ | Strong |
|  | CTS - Intensity | 3.71 (0.43) | 3.48 (0.59) | 3.71 (0.68) | .120 | 0.082 | H_0_ | Strong |
|  | CTS - Mood | 2.77 (0.51) | 2.68 (0.62) | 2.74 (0.78) | .925 | 0.014 | H_0_ | Strong |
|  | CTS - Persistence | 3.32 (0.75) | 3.05 (0.80) | 3.13 (0.82) | .565 | 0.041 | H_0_ | Strong |
|  | CTS - Distractibility | 2.05 (0.51) | 2.19 (0.62) | 2.28 (0.69) | .660 | 0.010 | H_0_ | Strong |
|  | CTS - Threshold | 3.83 (0.48) | 3.89 (0.54) | 3.79 (0.62) | .708 | 0.037 | H_0_ | Strong |
|  |  | *n* = 7 | *n* = 27 | *n* = 23 |  |  |  |  |
|  | ISP2 – Total Score | 55.71 (7.02) | 52.59 (8.06) | 54.74 (8.00) | .507 | 0.033 | H_0_ | Strong |
| 12 months |  | *n* = 14 | *n* = 37 | *n* = 22 |  |  |  |  |
|  | CTS - Activity | 4.31 (0.60) | 3.81 (0.52) | 3.82 (0.62) | .035 | 0.318 | H_0_ | Inconclusive |
|  | CTS - Rhythmicity | 2.56 (0.61) | 2.67 (0.94) | 2.64 (0.68) | .946 | 0.009 | H_0_ | Strong |
|  | CTS - Approach | 2.91 (0.81) | 3.09 (0.97) | 2.87 (0.54) | .464 | 0.034 | H_0_ | Strong |
|  | CTS - Adaptability | 3.70 (0.72) | 3.44 (0.84) | 3.52 (0.59) | .536 | 0.008 | H_0_ | Strong |
|  | CTS - Intensity | 3.74 (0.40) | 3.89 (0.52) | 3.72 (0.65) | .467 | 0.038 | H_0_ | Strong |
|  | CTS - Mood | 3.46 (0.61) | 3.05 (0.59) | 3.19 (0.68) | .067 | 0.153 | H_0_ | Moderate |
|  | CTS - Persistence | 4.00 (0.56) | 3.69 (0.58) | 3.79 (0.77) | .242 | 0.020 | H_0_ | Strong |
|  | CTS - Distractibility | 4.39 (0.42) | 4.40 (0.60) | 4.44 (0.53) | .707 | 0.033 | H_0_ | Strong |
|  | CTS - Threshold | 3.71 (0.56) | 3.55 (0.70) | 3.54 (0.74) | .434 | 0.042 | H_0_ | Strong |
|  |  | *n* = 13 | *n* = 36 | *n* = 21 |  |  |  |  |
|  | TSP2 - General Processing | 18.69 (3.99) | 15.81 (5.87) | 16.71 (5.39) | .237 | 0.017 | H_0_ | Strong |
|  | TSP2 - Auditory Processing | 11.23 (2.42) | 10.03 (3.95) | 10.52 (2.73) | .410 | 0.029 | H_0_ | Strong |
|  | TSP2 - Visual Processing | 15.15 (4.30) | 17.60 (2.95) | 17.05 (3.38) | .079 | 0.068 | H_0_ | Strong |
|  | TSP2 - Touch Processing | 10.31 (1.89) | 10.78 (3.06) | 9.71 (2.85) | .228 | 0.144 | H_0_ | Moderate |
|  | TSP2 - Movement Processing | 18.85 (1.95) | 18.00 (3.14) | 18.48 (2.71) | .595 | 0.033 | H_0_ | Strong |
|  | TSP2 - Oral Processing | 13.46 (3.04) | 12.03 (4.06) | 13.05 (3.79) | .416 | 0.097 | H_0_ | Strong |
|  | TSP2 - Behavioural Processing | 11.31 (3.59) | 11.06 (3.68) | 11.62 (3.49) | .930 | 0.025 | H_0_ | Strong |
|  | TSP2 - Seeking/Seeker Quadrant | 28.85 (3.67) | 30.50 (4.40) | 30.67 (3.65) | .323 | 0.069 | H_0_ | Strong |
|  | TSP2 - Avoiding/Avoider Quadrant | 16.85 (3.56) | 15.68 (4.96) | 15.90 (3.06) | .459 | 0.038 | H_0_ | Strong |
|  | TSP2 - Sensitivity/Sensor Quadrant | 26.33 (5.10) | 24.34 (6.36) | 24.19 (6.79) | .407 | 0.119 | H_0_ | Moderate |
|  | TSP2 – Registration/Bystander Quadrant | 16.15 (2.48) | 15.35 (4.73) | 16.43 (3.44) | .654 | 0.011 | H_0_ | Strong |

| **Table S5.** Comparison of the distribution of temperament and sensory processing scores between infants born to mothers with mild, moderate, and severe asthma. Chi-square tests were used to determine significant differences between the three groups, and *p*-values are reported here. Bayesian analyses were also conducted to provide the direction and strength of evidence. | | | | | | | | | | |
| --- | --- | --- | --- | --- | --- | --- | --- | --- | --- | --- |
| Age Group | Asthma Severity Group | <2 SD  Count (%) | < 1 SD  Count (%) | Within 1 SD  Count (%) | > 1 SD  Count (%) | > 2 SD  Count (%) | *p*-value (α = 0.006) | BF_10_ | Direction of Evidence | Strength of Evidence |
| 6 weeks |  |  |  |  |  |  |  |  |  |  |
| CTS - Activity | Mild | 4 (5.1) | 4 (5.1) | 56 (71.8) | 13 (16.7) | 1 (1.3) | .971 | 3.264 | H_a_ | Moderate |
|  | Moderate | 0 (0.0) | 1 (6.3) | 12 (75.0) | 3 (18.8) | 0 (0.0) |  |  |  |  |
|  | Severe | 1 (3.0) | 3 (9.1) | 24 (72.7) | 5 (15.2) | 0 (0.0) |  |  |  |  |
|  |  |  |  |  |  |  |  |  |  |  |
| CTS - Rhythmicity | Mild | 2 (2.4) | 2 (2.4) | 57 (67.1) | 15 (17.6) | 9 (10.6) | .076 | 0.024 | H_0_ | Strong |
|  | Moderate | 1 (5.6) | 0 (0.0) | 10 (55.6) | 4 (22.2) | 3 (16.7) |  |  |  |  |
|  | Severe | 1 (2.8) | 6 (16.7) | 21 (58.3) | 7 (19.4) | 1 (2.8) |  |  |  |  |
|  |  |  |  |  |  |  |  |  |  |  |
| CTS - Approach | Mild | 3 (3.6) | 14 (16.7) | 52 (61.9) | 13 (15.5) | 2 (2.4) | .770 | 2.561 | H_a_ | Inconclusive |
|  | Moderate | 2 (11.1) | 2 (11.1) | 11 (61.1) | 3 (16.7) | 0 (0.0) |  |  |  |  |
|  | Severe | 2 (5.3) | 7 (18.4) | 26 (68.4) | 3 (7.9) | 0 (0.0) |  |  |  |  |
|  |  |  |  |  |  |  |  |  |  |  |
| CTS - Adaptability | Mild | 1 (1.3) | 12 (16.0) | 54 (72.0) | 7 (9.3) | 1 (1.3) | .195 | 0.002 | H_0_ | Strong |
|  | Moderate | 0 (0.0) | 6 (37.5) | 8 (50.0) | 1 (6.3) | 1 (6.3) |  |  |  |  |
|  | Severe | 2 (5.6) | 10 (27.8) | 23 (63.9) | 1 (2.8) | 0 (0.0) |  |  |  |  |
|  |  |  |  |  |  |  |  |  |  |  |
| CTS - Intensity | Mild | 5 (6.0) | 21 (25.0) | 43 (51.2) | 12 (14.3) | 3 (3.6) | .784 | 0.001 | H_0_ | Strong |
|  | Moderate | 1 (5.6) | 5 (27.8) | 8 (44.4) | 3 (16.7) | 1 (5.6) |  |  |  |  |
|  | Severe | 3 (8.3) | 5 (13.9) | 20 (55.6) | 8 (22.2) | 0 (0.0) |  |  |  |  |
|  |  |  |  |  |  |  |  |  |  |  |
| CTS - Mood | Mild | 3 (3.6) | 20 (24.1) | 51 (61.4) | 6 (7.2) | 3 (3.6) | .543 | 7.966 | H_a_ | Moderate |
|  | Moderate | 1 (5.6) | 3 (16.7) | 12 (66.7) | 2 (11.1) | 0 (0.0) |  |  |  |  |
|  | Severe | 5 (13.9) | 8 (22.2) | 20 (55.6) | 3 (8.3) | 0 (0.0) |  |  |  |  |
|  |  |  |  |  |  |  |  |  |  |  |
| CTS - Persistence | Mild | 3 (3.7) | 18 (22.2) | 38 (46.9) | 17 (21.0) | 5 (6.2) | .490 | 0.004 | H_0_ | Strong |
|  | Moderate | 0 (0.0) | 2 (11.1) | 13 (72.2) | 2 (11.1) | 1 (5.6) |  |  |  |  |
|  | Severe | 2 (5.6) | 10 (27.8) | 16 (44.4) | 4 (11.1) | 4 (11.1) |  |  |  |  |
|  |  |  |  |  |  |  |  |  |  |  |
| CTS - Distractibility | Mild | 11 (13.3) | 21 (25.3) | 41 (49.4) | 7 (8.4) | 3 (3.6) | .891 | 5.106 | H_a_ | Moderate |
|  | Moderate | 4 (22.2) | 3 (16.7) | 10 (55.6) | 1 (5.6) | 0 (0.0) |  |  |  |  |
|  | Severe | 7 (20.0) | 8 (22.9) | 18 (51.4) | 1 (2.9) | 1 (2.9) |  |  |  |  |
|  |  |  |  |  |  |  |  |  |  |  |
| CTS - Threshold | Mild | 5 (6.0) | 4 (4.8) | 59 (71.1) | 11 (13.3) | 4 (4.8) | .121 | 0.025 | H_0_ | Strong |
|  | Moderate | 1 (5.6) | 0 (0.0) | 12 (66.7) | 5 (27.8) | 0 (0.0) |  |  |  |  |
|  | Severe | 3 (8.1) | 5 (13.5) | 16 (43.2) | 10 (27.0) | 3 (8.1) |  |  |  |  |
|  |  |  |  |  |  |  |  |  |  |  |
| ISP2 – Total Score | Mild | 0 (0.0) | 1 (1.7) | 45 (77.6) | 12 (20.7) | 0 (0.0) | .374 | 2.663 | H_a_ | Inconclusive |
|  | Moderate | 0 (0.0) | 0 (0.0) | 14 (100.0) | 0 (0.0) | 0 (0.0) |  |  |  |  |
|  | Severe | 0 (0.0) | 0 (0.0) | 20 (83.3) | 4 (16.7) | 0 (0.0) |  |  |  |  |
| 6 months |  |  |  |  |  |  |  |  |  |  |
| CTS - Activity | Mild | 1 (1.9) | 9 (17.3) | 41 (78.8) | 1 (1.9) | 0 (0.0) | .319 | 9.056 | H_a_ | Moderate |
|  | Moderate | 0 (0.0) | 0 (0.0) | 10 (90.9) | 1 (9.1) | 0 (0.0) |  |  |  |  |
|  | Severe | 1 (5.6) | 2 (11.1) | 14 (77.8) | 0 (0.0) | 1 (5.6) |  |  |  |  |
|  |  |  |  |  |  |  |  |  |  |  |
| CTS - Rhythmicity | Mild | 0 (0.0) | 4 (7.8) | 25 (49.0) | 14 (27.5) | 8 (15.7) | .180 | 0.212 | H_0_ | Moderate |
|  | Moderate | 0 (0.0) | 3 (27.3) | 3 (27.3) | 3 (27.3) | 2 (18.2) |  |  |  |  |
|  | Severe | 0 (0.0) | 0 (0.0) | 11 (61.1) | 6 (33.3) | 1 (5.6) |  |  |  |  |
|  |  |  |  |  |  |  |  |  |  |  |
| CTS - Approach | Mild | 0 (0.0) | 2 (4.1) | 40 (81.6) | 7 (14.3) | 0 (0.0) | .740 | 0.001 | H_0_ | Strong |
|  | Moderate | 0 (0.0) | 1 (9.1) | 7 (63.6) | 3 (27.3) | 0 (0.0) |  |  |  |  |
|  | Severe | 0 (0.0) | 1 (5.6) | 13 (72.2) | 4 (22.2) | 0 (0.0) |  |  |  |  |
|  |  |  |  |  |  |  |  |  |  |  |
| CTS - Adaptability | Mild | 0 (0.0) | 3 (5.9) | 42 (82.4) | 4 (7.8) | 2 (3.9) | .454 | 0.030 | H_0_ | Strong |
|  | Moderate | 0 (0.0) | 2 (18.2) | 7 (63.6) | 1 (9.1) | 1 (9.1) |  |  |  |  |
|  | Severe | 0 (0.0) | 2 (11.1) | 11 (61.1) | 4 (22.2) | 1 (5.6) |  |  |  |  |
|  |  |  |  |  |  |  |  |  |  |  |
| CTS - Intensity | Mild | 0 (0.0) | 4 (7.7) | 40 (76.9) | 8 (15.4) | 0 (0.0) | .531 | 0.006 | H_0_ | Strong |
|  | Moderate | 0 (0.0) | 0 (0.0) | 8 (72.7) | 2 (18.2) | 1 (9.1) |  |  |  |  |
|  | Severe | 0 (0.0) | 1 (5.6) | 14 (77.8) | 2 (11.1) | 1 (5.6) |  |  |  |  |
|  |  |  |  |  |  |  |  |  |  |  |
| CTS - Mood | Mild | 1 (1.9) | 10 (19.2) | 34 (65.4) | 7 (13.5) | 0 (0.0) | .485 | 0.022 | H_0_ | Strong |
|  | Moderate | 1 (9.1) | 3 (27.3) | 4 (36.4) | 3 (27.3) | 0 (0.0) |  |  |  |  |
|  | Severe | 0 (0.0) | 4 (22.2) | 12 (66.7) | 2 (11.1) | 0 (0.0) |  |  |  |  |
|  |  |  |  |  |  |  |  |  |  |  |
| CTS - Persistence | Mild | 1 (1.9) | 7 (13.5) | 35 (67.3) | 8 (15.4) | 1 (1.9) | .940 | 4.299 | H_a_ | Moderate |
|  | Moderate | 0 (0.0) | 1 (9.1) | 8 (72.7) | 2 (18.2) | 0 (0.0) |  |  |  |  |
|  | Severe | 0 (0.0) | 2 (10.5) | 11 (57.9) | 5 (26.3) | 1 (5.3) |  |  |  |  |
|  |  |  |  |  |  |  |  |  |  |  |
| CTS - Distractibility | Mild | 1 (1.9) | 12 (23.1) | 34 (65.4) | 5 (9.6) | 0 (0.0) | .426 | 0.007 | H_0_ | Strong |
|  | Moderate | 0 (0.0) | 2 (18.2) | 6 (54.5) | 2 (18.2) | 1 (9.1) |  |  |  |  |
|  | Severe | 0 (0.0) | 3 (15.8) | 10 (52.6) | 5 (26.3) | 1 (5.3) |  |  |  |  |
|  |  |  |  |  |  |  |  |  |  |  |
| CTS - Threshold | Mild | 0 (0.0) | 4 (8.0) | 42 (84.0) | 4 (8.0) | 0 (0.0) | .280 | 1.823 | H_a_ | Inconclusive |
|  | Moderate | 0 (0.0) | 1 (9.1) | 9 (81.8) | 0 (0.0) | 1 (9.1) |  |  |  |  |
|  | Severe | 0 (0.0) | 1 (5.6) | 15 (83.3) | 2 (11.1) | 0 (0.0) |  |  |  |  |
|  |  |  |  |  |  |  |  |  |  |  |
| ISP2 – Total Score | Mild | 0 (0.0) | 0 (0.0) | 29 (82.9) | 5 (14.3) | 1 (2.9) | .552 | 0.018 | H_0_ | Strong |
|  | Moderate | 0 (0.0) | 0 (0.0) | 9 (90.0) | 1 (10.0) | 0 (0.0) |  |  |  |  |
|  | Severe | 0 (0.0) | 0 (0.0) | 10 (90.9) | 0 (0.0) | 1 (9.1) |  |  |  |  |
| 12 months |  |  |  |  |  |  |  |  |  |  |
| CTS - Activity | Mild | 0 (0.0) | 6 (14.0) | 35 (81.4) | 2 (4.7) | 0 (0.0) | .067 | 0.057 | H_0_ | Strong |
|  | Moderate | 1 (8.3) | 0 (0.0) | 11 (91.7) | 0 (0.0) | 0 (0.0) |  |  |  |  |
|  | Severe | 0 (0.0) | 4 (36.4) | 6 (54.5) | 1 (9.1) | 0 (0.0) |  |  |  |  |
|  |  |  |  |  |  |  |  |  |  |  |
| CTS - Rhythmicity | Mild | 0 (0.0) | 5 (11.1) | 32 (71.1) | 5 (11.1) | 3 (6.7) | .755 | 0.006 | H_0_ | Strong |
|  | Moderate | 0 (0.0) | 1 (7.7) | 9 (69.2) | 2 (15.4) | 1 (7.7) |  |  |  |  |
|  | Severe | 0 (0.0) | 0 (0.0) | 12 (92.3) | 1 (7.7) | 0 (0.0) |  |  |  |  |
|  |  |  |  |  |  |  |  |  |  |  |
| CTS - Approach | Mild | 0 (0.0) | 3 (6.7) | 37 (82.2) | 4 (8.9) | 1 (2.2) | .615 | 0.001 | H_0_ | Strong |
|  | Moderate | 0 (0.0) | 0 (0.0) | 12 (92.3) | 0 (0.0) | 1 (7.7) |  |  |  |  |
|  | Severe | 0 (0.0) | 0 (0.0) | 11 (91.7) | 1 (8.3) | 0 (0.0) |  |  |  |  |
|  |  |  |  |  |  |  |  |  |  |  |
| CTS - Adaptability | Mild | 0 (0.0) | 3 (7.1) | 30 (71.4) | 9 (21.4) | 0 (0.0) | .139 | 0.229 | H_0_ | Moderate |
|  | Moderate | 0 (0.0) | 4 (30.8) | 8 (61.5) | 1 (7.7) | 0 (0.0) |  |  |  |  |
|  | Severe | 0 (0.0) | 1 (8.3) | 10 (83.3) | 1 (8.3) | 0 (0.0) |  |  |  |  |
|  |  |  |  |  |  |  |  |  |  |  |
| CTS - Intensity | Mild | 0 (0.0) | 8 (18.2) | 33 (75.0) | 3 (6.8) | 0 (0.0) | .976 | 9.914 | H_a_ | Moderate |
|  | Moderate | 0 (0.0) | 2 (15.4) | 10 (76.9) | 1 (7.7) | 0 (0.0) |  |  |  |  |
|  | Severe | 0 (0.0) | 3 (25.0) | 8 (66.7) | 1 (8.3) | 0 (0.0) |  |  |  |  |
|  |  |  |  |  |  |  |  |  |  |  |
| CTS - Mood | Mild | 1 (2.2) | 2 (4.4) | 28 (62.2) | 13 (28.9) | 1 (2.2) | .342 | 0.013 | H_0_ | Strong |
|  | Moderate | 0 (0.0) | 2 (15.4) | 8 (61.5) | 2 (15.4) | 1 (7.7) |  |  |  |  |
|  | Severe | 0 (0.0) | 1 (8.3) | 11 (91.7) | 0 (0.0) | 0 (0.0) |  |  |  |  |
|  |  |  |  |  |  |  |  |  |  |  |
| CTS - Persistence | Mild | 0 (0.0) | 1 (2.2) | 37 (82.2) | 5 (11.1) | 2 (4.4) | .408 | 0.008 | H_0_ | Strong |
|  | Moderate | 0 (0.0) | 0 (0.0) | 8 (66.7) | 4 (33.3) | 0 (0.0) |  |  |  |  |
|  | Severe | 0 (0.0) | 1 (9.1) | 8 (72.7) | 2 (18.2) | 0 (0.0) |  |  |  |  |
|  |  |  |  |  |  |  |  |  |  |  |
| CTS - Distractibility | Mild | 0 (0.0) | 4 (8.9) | 36 (80.0) | 5 (11.1) | 0 (0.0) | .148 | 0.065 | H_0_ | Strong |
|  | Moderate | 0 (0.0) | 3 (23.1) | 10 (76.9) | 0 (0.0) | 0 (0.0) |  |  |  |  |
|  | Severe | 0 (0.0) | 0 (0.0) | 12 (100.0) | 0 (0.0) | 0 (0.0) |  |  |  |  |
|  |  |  |  |  |  |  |  |  |  |  |
| CTS - Threshold | Mild | 1 (2.3) | 7 (15.9) | 30 (68.2) | 6 (13.6) | 0 (0.0) | .435 | 0.007 | H_0_ | Strong |
|  | Moderate | 0 (0.0) | 0 (0.0) | 12 (92.3) | 1 (7.7) | 0 (0.0) |  |  |  |  |
|  | Severe | 0 (0.0) | 1 (8.3) | 11 (91.7) | 0 (0.0) | 0 (0.0) |  |  |  |  |
|  |  |  |  |  |  |  |  |  |  |  |
| TSP2 - General | Mild | 1 (2.4) | 1 (2.4) | 35 (83.3) | 4 (9.5) | 1 (2.4) | .854 | 8.662 | H_a_ | Moderate |
|  | Moderate | 0 (0.0) | 0 (0.0) | 12 (92.3) | 0 (0.0) | 1 (7.7) |  |  |  |  |
|  | Severe | 0 (0.0) | 0 (0.0) | 12 (92.3) | 1 (7.7) | 0 (0.0) |  |  |  |  |
|  |  |  |  |  |  |  |  |  |  |  |
| TSP2 - Auditory | Mild | 1 (2.4) | 0 (0.0) | 37 (90.2) | 3 (7.3) | 0 (0.0) | .451 | 5.035 | H_a_ | Moderate |
|  | Moderate | 0 (0.0) | 0 (0.0) | 12 (92.3) | 0 (0.0) | 1 (7.7) |  |  |  |  |
|  | Severe | 0 (0.0) | 0 (0.0) | 12 (92.3) | 1 (7.7) | 0 (0.0) |  |  |  |  |
|  |  |  |  |  |  |  |  |  |  |  |
| TSP2 - Visual | Mild | 0 (0.0) | 1 (2.4) | 31 (73.8) | 10 (23.8) | 0 (0.0) | .137 | 0.005 | H_0_ | Strong |
|  | Moderate | 0 (0.0) | 2 (16.7) | 8 (66.7) | 2 (16.7) | 0 (0.0) |  |  |  |  |
|  | Severe | 0 (0.0) | 0 (0.0) | 12 (92.3) | 1 (7.7) | 0 (0.0) |  |  |  |  |
|  |  |  |  |  |  |  |  |  |  |  |
| TSP2 - Touch | Mild | 0 (0.0) | 0 (0.0) | 35 (83.3) | 7 (16.7) | 0 (0.0) | .291 | 0.013 | H_0_ | Strong |
|  | Moderate | 0 (0.0) | 0 (0.0) | 10 (76.9) | 2 (15.4) | 1 (7.7) |  |  |  |  |
|  | Severe | 0 (0.0) | 0 (0.0) | 12 (92.3) | 1 (7.7) | 0 (0.0) |  |  |  |  |
|  |  |  |  |  |  |  |  |  |  |  |
| TSP2 - Movement | Mild | 0 (0.0) | 0 (0.0) | 29 (69.0) | 13 (31.0) | 0 (0.0) | .043 | 0.323 | H_0_ | Inconclusive |
|  | Moderate | 1 (7.7) | 0 (0.0) | 8 (61.5) | 4 (30.8) | 0 (0.0) |  |  |  |  |
|  | Severe | 0 (0.0) | 0 (0.0) | 13 (100.0) | 0 (0.0) | 0 (0.0) |  |  |  |  |
|  |  |  |  |  |  |  |  |  |  |  |
| TSP2 - Oral | Mild | 0 (0.0) | 0 (0.0) | 31 (75.6) | 8 (19.5) | 2 (4.9) | .752 | 0.014 | H_0_ | Strong |
|  | Moderate | 0 (0.0) | 0 (0.0) | 11 (84.6) | 1 (7.7) | 1 (7.7) |  |  |  |  |
|  | Severe | 0 (0.0) | 0 (0.0) | 11 (84.6) | 2 (15.4) | 0 (0.0) |  |  |  |  |
|  |  |  |  |  |  |  |  |  |  |  |
| TSP2 - Behavioural | Mild | 0 (0.0) | 1 (2.5) | 31 (77.5) | 5 (12.5) | 3 (7.5) | .580 | 0.004 | H_0_ | Strong |
|  | Moderate | 0 (0.0) | 1 (7.7) | 10 (76.9) | 1 (7.7) | 1 (7.7) |  |  |  |  |
|  | Severe | 0 (0.0) | 0 (0.0) | 13 (100.0) | 0 (0.0) | 0 (0.0) |  |  |  |  |
|  |  |  |  |  |  |  |  |  |  |  |
| TSP2 - Seeking/Seeker | Mild | 0 (0.0) | 1 (2.4) | 27 (64.3) | 14 (33.3) | 0 (0.0) | .068 | 0.039 | H_0_ | Strong |
|  | Moderate | 1 (7.7) | 0 (0.0) | 10 (76.9) | 2 (15.4) | 0 (0.0) |  |  |  |  |
|  | Severe | 0 (0.0) | 0 (0.0) | 13 (100.0) | 0 (0.0) | 0 (0.0) |  |  |  |  |
|  |  |  |  |  |  |  |  |  |  |  |
| TSP2 - Avoiding/  Avoider | Mild | 0 (0.0) | 2 (5.0) | 34 (85.0) | 4 (10.0) | 0 (0.0) | .221 | 0.001 | H_0_ | Strong |
|  | Moderate | 0 (0.0) | 0 (0.0) | 12 (92.3) | 0 (0.0) | 1 (7.7) |  |  |  |  |
|  | Severe | 0 (0.0) | 0 (0.0) | 13 (100.0) | 0 (0.0) | 0 (0.0) |  |  |  |  |
|  |  |  |  |  |  |  |  |  |  |  |
| TSP2 - Sensitivity/  Sensor | Mild | 0 (0.0) | 0 (0.0) | 27 (71.1) | 8 (21.1) | 3 (7.9) | .821 | 0.017 | H_0_ | Strong |
|  | Moderate | 0 (0.0) | 0 (0.0) | 10 (76.9) | 2 (15.4) | 1 (7.7) |  |  |  |  |
|  | Severe | 0 (0.0) | 0 (0.0) | 11 (84.6) | 2 (15.4) | 0 (0.0) |  |  |  |  |
|  |  |  |  |  |  |  |  |  |  |  |
| TSP2 – Registration/  Bystander | Mild | 0 (0.0) | 2 (4.8) | 36 (85.7) | 4 (9.5) | 0 (0.0) | .194 | 0.002 | H_0_ | Strong |
|  | Moderate | 0 (0.0) | 0 (0.0) | 10 (90.9) | 0 (0.0) | 1 (9.1) |  |  |  |  |
|  | Severe | 0 (0.0) | 0 (0.0) | 13 (100.0) | 0 (0.0) | 0 (0.0) |  |  |  |  |

| **Table S6.** Comparison of the distribution of temperament and sensory processing scores between infants born to mothers with well-controlled, partly-controlled, and uncontrolled asthma. Chi-square tests were used to determine significant differences between the three groups, and *p*-values are reported here. Bayesian analyses were also conducted to provide the direction and strength of evidence. | | | | | | | | | | |
| --- | --- | --- | --- | --- | --- | --- | --- | --- | --- | --- |
| Age Group | Asthma Control Group | <2 SD  Count (%) | < 1 SD  Count (%) | Within 1 SD  Count (%) | > 1 SD  Count (%) | > 2 SD  Count (%) | *p*-value (α = 0.006) | BF_10_ | Direction of Evidence | Strength of Evidence |
| 6 weeks |  |  |  |  |  |  |  |  |  |  |
| CTS - Activity | Well | 1 (3.8) | 1 (3.8) | 20 (76.9) | 4 (15.4) | 0 (0.0) | .508 | 2.719 | H_a_ | Inconclusive |
|  | Partly | 4 (6.7) | 3 (5.0) | 45 (75.0) | 7 (11.7) | 1 (1.7) |  |  |  |  |
|  | Un | 0 (0.0) | 4 (9.5) | 27 (64.3) | 10 (23.8) | 1 (2.4) |  |  |  |  |
|  |  |  |  |  |  |  |  |  |  |  |
| CTS - Rhythmicity | Well | 1 (3.3) | 3 (10.0) | 19 (63.3) | 4 (13.3) | 3 (10.0) | .929 | 1.474 | H_a_ | Inconclusive |
|  | Partly | 2 (3.0) | 2 (3.0) | 44 (66.7) | 12 (18.2) | 6 (9.1) |  |  |  |  |
|  | Un | 1 (2.3) | 3 (6.8) | 26 (59.1) | 10 (22.7) | 4 (9.1) |  |  |  |  |
|  |  |  |  |  |  |  |  |  |  |  |
| CTS - Approach | Well | 3 (10.3) | 4 (13.8) | 16 (55.2) | 6 (20.7) | 0 (0.0) | .416 | 8.235 | H_a_ | Moderate |
|  | Partly | 4 (5.9) | 13 (19.1) | 44 (64.7) | 6 (8.8) | 1 (1.5) |  |  |  |  |
|  | Un | 0 (0.0) | 6 (13.6) | 31 (70.5) | 6 (13.6) | 1 (2.3) |  |  |  |  |
|  |  |  |  |  |  |  |  |  |  |  |
| CTS - Adaptability | Well | 1 (3.8) | 4 (15.4) | 20 (76.9) | 1 (3.8) | 0 (0.0) | .009 | 0.087 | H_0_ | Strong |
|  | Partly | 1 (1.7) | 15 (25.0) | 42 (70.0) | 0 (0.0) | 2 (3.3) |  |  |  |  |
|  | Un | 1 (2.4) | 9 (22.0) | 22 (53.7) | 9 (22.0) | 0 (0.0) |  |  |  |  |
|  |  |  |  |  |  |  |  |  |  |  |
| CTS - Intensity | Well | 2 (6.7) | 6 (20.0) | 15 (50.0) | 6 (20.0) | 1 (3.3) | .985 | 1.454 | H_a_ | Inconclusive |
|  | Partly | 5 (7.6) | 15 (22.7) | 35 (53.0) | 10 (15.2) | 1 (1.5) |  |  |  |  |
|  | Un | 2 (4.7) | 10 (23.3) | 23 (53.5) | 6 (14.0) | 2 (4.7) |  |  |  |  |
|  |  |  |  |  |  |  |  |  |  |  |
| CTS - Mood | Well | 1 (3.6) | 5 (17.9) | 20 (71.4) | 1 (3.6) | 1 (3.6) | .180 | 0.004 | H_0_ | Strong |
|  | Partly | 5 (7.6) | 19 (28.8) | 39 (59.1) | 3 (4.5) | 0 (0.0) |  |  |  |  |
|  | Un | 3 (6.8) | 7 (15.9) | 25 (56.8) | 7 (15.9) | 2 (4.5) |  |  |  |  |
|  |  |  |  |  |  |  |  |  |  |  |
| CTS - Persistence | Well | 2 (7.1) | 5 (17.9) | 13 (46.4) | 4 (14.3) | 4 (14.3) | .139 | 0.025 | H_0_ | Strong |
|  | Partly | 2 (3.1) | 15 (23.4) | 38 (59.4) | 6 (9.4) | 3 (4.7) |  |  |  |  |
|  | Un | 1 (2.3) | 9 (20.5) | 18 (40.9) | 13 (29.5) | 3 (6.8) |  |  |  |  |
|  |  |  |  |  |  |  |  |  |  |  |
| CTS - Distractibility | Well | 6 (20.7) | 9 (31.0) | 10 (34.5) | 3 (10.3) | 1 (3.4) | .221 | 0.014 | H_0_ | Strong |
|  | Partly | 1117.2) | 17 (26.6) | 32 (50.0) | 4 (6.3) | 0 (0.0) |  |  |  |  |
|  | Un | 5 (11.6) | 6 (14.0) | 26 (60.5) | 3 (7.0) | 3 (7.0) |  |  |  |  |
|  |  |  |  |  |  |  |  |  |  |  |
| CTS - Threshold | Well | 1 (3.6) | 2 (7.1) | 19 (67.9) | 4 (14.3) | 2 (7.1) | .978 | 1.168 | H_a_ | Inconclusive |
|  | Partly | 6 (9.0) | 4 (6.0) | 41 (61.2) | 13 (19.4) | 3 (4.5) |  |  |  |  |
|  | Un | 2 (4.5) | 3 (6.8) | 28 (63.6) | 8 (18.2) | 3 (6.8) |  |  |  |  |
|  |  |  |  |  |  |  |  |  |  |  |
| ISP2 – Total Score | Well | 0 (0.0) | 0 (0.0) | 15 (93.8) | 1 (6.3) | 0 (0.0) | .573 | 7.128 | H_a_ | Moderate |
|  | Partly | 0 (0.0) | 1 (2.1) | 39 (83.0) | 7 (14.9) | 0 (0.0) |  |  |  |  |
|  | Un | 0 (0.0) | 0 (0.0) | 26 (78.8) | 7 (21.2) | 0 (0.0) |  |  |  |  |
| 6 months |  |  |  |  |  |  |  |  |  |  |
| CTS - Activity | Well | 0 (0.0) | 1 (6.7) | 14 (93.3) | 0 (0.0) | 0 (0.0) | .347 | 3.004 | H_a_ | Moderate |
|  | Partly | 0 (0.0) | 6 (14.3) | 35 (83.3) | 1 (2.4) | 0 (0.0) |  |  |  |  |
|  | Un | 2 (8.0) | 4 (16.0) | 17 (68.0) | 1 (4.0) | 1 (4.0) |  |  |  |  |
|  |  |  |  |  |  |  |  |  |  |  |
| CTS - Rhythmicity | Well | 0 (0.0) | 2 (13.3) | 9 (60.0) | 3 (20.0) | 1 (6.7) | .741 | 0.017 | H_0_ | Strong |
|  | Partly | 0 (0.0) | 3 (7.3) | 17 (41.5) | 14 (34.1) | 7 (17.1) |  |  |  |  |
|  | Un | 0 (0.0) | 2 (8.0) | 14 (56.0) | 6 (24.0) | 3 (12.0) |  |  |  |  |
|  |  |  |  |  |  |  |  |  |  |  |
| CTS - Approach | Well | 0 (0.0) | 0 (0.0) | 14 (93.3) | 1 (6.7) | 0 (0.0) | .470 | 6.618 | H_a_ | Moderate |
|  | Partly | 0 (0.0) | 2 (5.1) | 28 (71.8) | 9 (23.1) | 0 (0.0) |  |  |  |  |
|  | Un | 0 (0.0) | 2 (8.0) | 19 (76.0) | 4 (16.0) | 0 (0.0) |  |  |  |  |
|  |  |  |  |  |  |  |  |  |  |  |
| CTS - Adaptability | Well | 0 (0.0) | 1 (6.7) | 14 (93.3) | 0 (0.0) | 0 (0.0) | .528 | 0.006 | H_0_ | Strong |
|  | Partly | 0 (0.0) | 4 (9.8) | 27 (65.9) | 7 (17.1) | 3 (7.3) |  |  |  |  |
|  | Un | 0 (0.0) | 2 (8.0) | 19 (76.0) | 3 (12.0) | 1 (4.0) |  |  |  |  |
|  |  |  |  |  |  |  |  |  |  |  |
| CTS - Intensity | Well | 0 (0.0) | 0 (0.0) | 13 (86.7) | 2 (13.3) | 0 (0.0) | .265 | 0.005 | H_0_ | Strong |
|  | Partly | 0 (0.0) | 4 (9.5) | 33 (78.6) | 5 (11.9) | 0 (0.0) |  |  |  |  |
|  | Un | 0 (0.0) | 1 (4.0) | 17 (68.0) | 5 (20.0) | 2 (8.0) |  |  |  |  |
|  |  |  |  |  |  |  |  |  |  |  |
| CTS - Mood | Well | 0 (0.0) | 3 (20.0) | 11 (73.3) | 1 (6.7) | 0 (0.0) | .434 | 0.015 | H_0_ | Strong |
|  | Partly | 1 (2.4) | 7 (16.7) | 29 (69.0) | 5 (11.9) | 0 (0.0) |  |  |  |  |
|  | Un | 1 (4.0) | 7 (28.0) | 11 (44.0) | 6 (24.0) | 0 (0.0) |  |  |  |  |
|  |  |  |  |  |  |  |  |  |  |  |
| CTS - Persistence | Well | 1 (6.7) | 0 (0.0) | 11 (73.3) | 3 (20.0) | 0 (0.0) | .443 | 9.832 | H_a_ | Moderate |
|  | Partly | 0 (0.0) | 7 (16.3) | 27 (62.8) | 8 (18.6) | 1 (2.3) |  |  |  |  |
|  | Un | 0 (0.0) | 3 (12.0) | 17 (68.0) | 4 (16.0) | 1 (4.0) |  |  |  |  |
|  |  |  |  |  |  |  |  |  |  |  |
| CTS - Distractibility | Well | 1 (6.7) | 2 (13.3) | 12 (80.0) | 0 (0.0) | 0 (0.0) | .200 | 0.006 | H_0_ | Strong |
|  | Partly | 0 (0.0) | 9 (20.9) | 27 (62.8) | 6 (14.0) | 1 (2.3) |  |  |  |  |
|  | Un | 0 (0.0) | 6 (24.0) | 12 (48.0) | 6 (24.0) | 1 (4.0) |  |  |  |  |
|  |  |  |  |  |  |  |  |  |  |  |
| CTS - Threshold | Well | 0 (0.0) | 1 (6.7) | 13 (86.7) | 1 (6.7) | 0 (0.0) | .909 | 2.275 | H_a_ | Inconclusive |
|  | Partly | 0 (0.0) | 3 (7.5) | 34 (85.0) | 2 (5.0) | 1 (2.5) |  |  |  |  |
|  | Un | 0 (0.0) | 2 (8.0) | 20 (80.0) | 3 (12.0) | 0 (0.0) |  |  |  |  |
|  |  |  |  |  |  |  |  |  |  |  |
| ISP2 – Total Score | Well | 0 (0.0) | 6 (85.7) | 1 (14.3) | 0 (0.0) | 0 (0.0) | .976 | 0.007 | H_0_ | Strong |
|  | Partly | 0 (0.0) | 23 (85.2) | 3 (11.1) | 1 (3.7) | 0 (0.0) |  |  |  |  |
|  | Un | 0 (0.0) | 20 (87.0) | 2 (8.7) | 1 (4.3) | 0 (0.0) |  |  |  |  |
| 12 months |  |  |  |  |  |  |  |  |  |  |
| CTS - Activity | Well | 0 (0.0) | 1 (7.1) | 11 (78.6) | 2 (14.3) | 0 (0.0) | .226 | 0.009 | H_0_ | Strong |
|  | Partly | 1 (2.9) | 4 (11.8) | 29 (85.3) | 0 (0.0) | 0 (0.0) |  |  |  |  |
|  | Un | 0 (0.0) | 5 (25.0) | 14 (70.0) | 1 (5.0) | 0 (0.0) |  |  |  |  |
|  |  |  |  |  |  |  |  |  |  |  |
| CTS - Rhythmicity | Well | 0 (0.0) | 0 (0.0) | 13 (92.9) | 1 (7.1) | 0 (0.0) | .365 | 0.011 | H_0_ | Strong |
|  | Partly | 0 (0.0) | 5 (13.5) | 23 (62.2) | 6 (16.2) | 3 (8.1) |  |  |  |  |
|  | Un | 0 (0.0) | 1 (4.5) | 18 (81.8) | 2 (9.1) | 1 (4.5) |  |  |  |  |
|  |  |  |  |  |  |  |  |  |  |  |
| CTS - Approach | Well | 0 (0.0) | 0 (0.0) | 12 (85.7) | 2 (14.3) | 0 (0.0) | .174 | 0.007 | H_0_ | Strong |
|  | Partly | 0 (0.0) | 3 (8.6) | 26 (74.3) | 4 (11.4) | 2 (5.7) |  |  |  |  |
|  | Un | 0 (0.0) | 0 (0.0) | 22 (100.0) | 0 (0.0) | 0 (0.0) |  |  |  |  |
|  |  |  |  |  |  |  |  |  |  |  |
| CTS - Adaptability | Well | 0 (0.0) | 1 (7.1) | 10 (71.4) | 3 (21.4) | 0 (0.0) | .465 | 0.055 | H_0_ | Strong |
|  | Partly | 0 (0.0) | 6 (18.8) | 20 (62.5) | 6 (18.8) | 0 (0.0) |  |  |  |  |
|  | Un | 0 (0.0) | 1 (4.5) | 18 (81.8) | 3 (13.6) | 0 (0.0) |  |  |  |  |
|  |  |  |  |  |  |  |  |  |  |  |
| CTS - Intensity | Well | 0 (0.0) | 2 (14.3) | 12 (85.7) | 0 (0.0) | 0 (0.0) | .201 | 0.003 | H_0_ | Strong |
|  | Partly | 0 (0.0) | 4 (11.4) | 27 (77.1) | 4 (11.4) | 0 (0.0) |  |  |  |  |
|  | Un | 0 (0.0) | 7 (31.8) | 14 (63.6) | 1 (4.5) | 0 (0.0) |  |  |  |  |
|  |  |  |  |  |  |  |  |  |  |  |
| CTS - Mood | Well | 0 (0.0) | 2 (14.3) | 6 (42.9) | 6 (42.9) | 0 (0.0) | .230 | 0.011 | H_0_ | Strong |
|  | Partly | 1 (2.9) | 1 (2.9) | 28 (80.0) | 4 (11.4) | 1 (2.9) |  |  |  |  |
|  | Un | 0 (0.0) | 2 (9.1) | 14 (63.6) | 5 (22.7) | 1 (4.5) |  |  |  |  |
|  |  |  |  |  |  |  |  |  |  |  |
| CTS - Persistence | Well | 0 (0.0) | 0 (0.0) | 9 (64.3) | 5 (35.7) | 0 (0.0) | .242 | 0.014 | H_0_ | Strong |
|  | Partly | 0 (0.0) | 1 (2.9) | 30 (88.2) | 2 (5.9) | 1 (2.9) |  |  |  |  |
|  | Un | 0 (0.0) | 1 (4.8) | 15 (71.4) | 4 (19.0) | 1 (4.8) |  |  |  |  |
|  |  |  |  |  |  |  |  |  |  |  |
| CTS - Distractibility | Well | 0 (0.0) | 1 (7.1) | 1 (92.9) | 0 (0.0) | 0 (0.0) | .579 | 0.013 | H_0_ | Strong |
|  | Partly | 0 (0.0) | 5 (13.9) | 28 (77.8) | 3 (8.3) | 0 (0.0) |  |  |  |  |
|  | Un | 0 (0.0) | 1 (4.5) | 19 (86.4) | 2 (9.1) | 0 (0.0) |  |  |  |  |
|  |  |  |  |  |  |  |  |  |  |  |
| CTS - Threshold | Well | 0 (0.0) | 1 (7.1) | 12 (85.7) | 1 (7.1) | 0 (0.0) | .936 | 8.252 | H_a_ | Moderate |
|  | Partly | 1 (2.9) | 4 (11.8) | 25 (73.5) | 4 (11.8) | 0 (0.0) |  |  |  |  |
|  | Un | 0 (0.0) | 3 (13.6) | 17 (77.3) | 2 (9.1) | 0 (0.0) |  |  |  |  |
|  |  |  |  |  |  |  |  |  |  |  |
| TSP2 - General | Well | 0 (0.0) | 0 (0.0) | 11 (84.6) | 2 (15.4) | 0 (0.0) | .595 | 1.713 | H_a_ | Inconclusive |
|  | Partly | 1 (2.8) | 0 (0.0) | 33 (91.7) | 1 (2.8) | 1 (2.8) |  |  |  |  |
|  | Un | 0 (0.0) | 1 (4.8) | 17 (81.0) | 2 (9.5) | 1 (4.8) |  |  |  |  |
|  |  |  |  |  |  |  |  |  |  |  |
| TSP2 - Auditory | Well | 0 (0.0) | 0 (0.0) | 12 (92.3) | 1 (7.7) | 0 (0.0) | .802 | 1.474 | H_a_ | Inconclusive |
|  | Partly | 1 (2.9) | 0 (0.0) | 32 (91.4) | 1 (2.9) | 1 (2.9) |  |  |  |  |
|  | Un | 0 (0.0) | 0 (0.0) | 19 (90.5) | 2 (9.5) | 0 (0.0) |  |  |  |  |
|  |  |  |  |  |  |  |  |  |  |  |
| TSP2 - Visual | Well | 0 (0.0) | 2 (15.4) | 10 (76.9) | 1 (7.7) | 0 (0.0) | .168 | 0.004 | H_0_ | Strong |
|  | Partly | 0 (0.0) | 0 (0.0) | 28 (80.0) | 7 (20.0) | 0 (0.0) |  |  |  |  |
|  | Un | 0 (0.0) | 1 (4.8) | 15 (71.4) | 5 (23.8) | 0 (0.0) |  |  |  |  |
|  |  |  |  |  |  |  |  |  |  |  |
| TSP2 - Touch | Well | 0 (0.0) | 0 (0.0) | 12 (92.3) | 1 (7.7) | 0 (0.0) | .606 | 0.005 | H_0_ | Strong |
|  | Partly | 0 (0.0) | 0 (0.0) | 28 (77.8) | 7 (19.4) | 1 (2.8) |  |  |  |  |
|  | Un | 0 (0.0) | 0 (0.0) | 19 (90.5) | 2 (9.5) | 0 (0.0) |  |  |  |  |
|  |  |  |  |  |  |  |  |  |  |  |
| TSP2 - Movement | Well | 0 (0.0) | 0 (0.0) | 11 (84.6) | 2 (15.4) | 0 (0.0) | .646 | 0.007 | H_0_ | Strong |
|  | Partly | 1 (2.8) | 0 (0.0) | 27 (75.0) | 8 (22.2) | 0 (0.0) |  |  |  |  |
|  | Un | 0 (0.0) | 0 (0.0) | 14 (66.7) | 7 (33.3) | 0 (0.0) |  |  |  |  |
|  |  |  |  |  |  |  |  |  |  |  |
| TSP2 - Oral | Well | 0 (0.0) | 0 (0.0) | 9 (69.2) | 4 (30.8) | 0 (0.0) | .549 | 0.020 | H_0_ | Strong |
|  | Partly | 0 (0.0) | 0 (0.0) | 29 ((82.9) | 4 (11.4) | 2 (5.7) |  |  |  |  |
|  | Un | 0 (0.0) | 0 (0.0) | 16 (76.2) | 4 (19.0) | 1 (4.8) |  |  |  |  |
|  |  |  |  |  |  |  |  |  |  |  |
| TSP2 - Behavioural | Well | 0 (0.0) | 0 (0.0) | 11 (84.6) | 1 (7.7) | 1 (7.7) | .399 | 0.006 | H_0_ | Strong |
|  | Partly | 0 (0.0) | 2 (5.9) | 28 (82.4) | 1 (2.9) | 3 (8.8) |  |  |  |  |
|  | Un | 0 (0.0) | 0 (0.0) | 16 (76.2) | 4 (19.0) | 1 (4.8) |  |  |  |  |
|  |  |  |  |  |  |  |  |  |  |  |
| TSP2 - Seeking/Seeker | Well | 0 (0.0) | 0 (0.0) | 12 (92.3) | 1 (7.7) | 0 (0.0) | .616 | 9.751 | H_a_ | Moderate |
|  | Partly | 1 (2.8) | 1 (2.8) | 24 (66.7) | 10 (27.8) | 0 (0.0) |  |  |  |  |
|  | Un | 0 (0.0) | 0 (0.0) | 16 (76.2) | 5 (23.8) | 0 (0.0) |  |  |  |  |
|  |  |  |  |  |  |  |  |  |  |  |
| TSP2 - Avoiding/Avoider | Well | 0 (0.0) | 0 (0.0) | 11 (84.6) | 2 (15.4) | 0 (0.0) | .360 | 0.001 | H_0_ | Strong |
|  | Partly | 0 (0.0) | 2 (5.9) | 29 (85.3) | 2 (5.9) | 1 (2.9) |  |  |  |  |
|  | Un | 0 (0.0) | 0 (0.0) | 21 (100.0) | 0 (0.0) | 0 (0.0) |  |  |  |  |
|  |  |  |  |  |  |  |  |  |  |  |
| TSP2 - Sensitivity/Sensor | Well | 0 (0.0) | 0 (0.0) | 8 (66.7) | 3 (25.0) | 1 (8.3) | .807 | 0.022 | H_0_ | Strong |
|  | Partly | 0 (0.0) | 0 (0.0) | 24 (75.0) | 7 (21.9) | 1 (3.1) |  |  |  |  |
|  | Un | 0 (0.0) | 0 (0.0) | 16 (76.2) | 3 (14.3) | 2 (9.5) |  |  |  |  |
|  |  |  |  |  |  |  |  |  |  |  |
| TSP2 – Registration/  Bystander | Well | 0 (0.0) | 0 (0.0) | 13 (100.0) | 0 (0.0) | 0 (0.0) | .614 | 3.768 | H_a_ | Moderate |
|  | Partly | 0 (0.0) | 2 (5.9) | 29 (85.3) | 2 (5.9) | 1 (2.9) |  |  |  |  |
|  | Un | 0 (0.0) | 0 (0.0) | 19 (90.5) | 2 (9.5) | 0 (0.0) |  |  |  |  |
